# Decoding Biomass-Sensing Regulons of *Clostridium thermocellum* Alternative Sigma-I Factors in a Heterologous *Bacillus subtilis* Host System

**DOI:** 10.1371/journal.pone.0146316

**Published:** 2016-01-05

**Authors:** Iván Muñoz-Gutiérrez, Lizett Ortiz de Ora, Inna Rozman Grinberg, Yuval Garty, Edward A. Bayer, Yuval Shoham, Raphael Lamed, Ilya Borovok

**Affiliations:** 1 Department of Molecular Microbiology and Biotechnology, Tel Aviv University, Tel Aviv, 69978, Israel; 2 Department of Biological Chemistry, The Weizmann Institute of Science, Rehovot, 76100, Israel; 3 Department of Biotechnology and Food Engineering, Technion-IIT, Haifa, 32000, Israel; University Paris South, FRANCE

## Abstract

The Gram-positive, anaerobic, cellulolytic, thermophile *Clostridium* (*Ruminiclostridium*) *thermocellum* secretes a multi-enzyme system called the cellulosome to solubilize plant cell wall polysaccharides. During the saccharolytic process, the enzymatic composition of the cellulosome is modulated according to the type of polysaccharide(s) present in the environment. *C*. *thermocellum* has a set of eight alternative RNA polymerase sigma (σ) factors that are activated in response to extracellular polysaccharides and share sequence similarity to the *Bacillus subtilis* σ^I^ factor. The aim of the present work was to demonstrate whether individual *C*. *thermocellum* σ^I^-like factors regulate specific cellulosomal genes, focusing on *C*. *thermocellum* σ^I6^ and σ^I3^ factors. To search for putative σ^I6^- and σ^I3^-dependent promoters, bioinformatic analysis of the upstream regions of the cellulosomal genes was performed. Because of the limited genetic tools available for *C*. *thermocellum*, the functionality of the predicted σ^I6^- and σ^I3^-dependent promoters was studied in *B*. *subtilis* as a heterologous host. This system enabled observation of the activation of 10 predicted σ^I6^-dependent promoters associated with the *C*. *thermocellum* genes: *sigI6* (itself, Clo1313_2778), *xyn11B* (Clo1313_0522), *xyn10D* (Clo1313_0177), *xyn10Z* (Clo1313_2635), *xyn10Y* (Clo1313_1305), *cel9V* (Clo1313_0349), *cseP* (Clo1313_2188), *sigI1* (Clo1313_2174), *cipA* (Clo1313_0627), and *rsgI5* (Clo1313_0985). Additionally, we observed the activation of 4 predicted σ^I3^-dependent promoters associated with the *C*. *thermocellum* genes: *sigI3* (itself, Clo1313_1911), *pl11* (Clo1313_1983), *ce12* (Clo1313_0693) and *cipA*. Our results suggest possible regulons of σ^I6^ and σ^I3^ in *C*. *thermocellum*, as well as the σ^I6^ and σ^I3^ promoter consensus sequences. The proposed -35 and -10 promoter consensus elements of σ^I6^ are CNNAAA and CGAA, respectively. Additionally, a less conserved CGA sequence next to the C in the -35 element and a highly conserved AT sequence three bases downstream of the -10 element were also identified as important nucleotides for promoter recognition. Regarding σ^I3^, the proposed -35 and -10 promoter consensus elements are CCCYYAAA and CGWA, respectively. The present study provides new clues for understanding these recently discovered alternative σ^I^ factors.

## Introduction

*Clostridium* (*Ruminiclostridium*) *thermocellum* is a Gram-positive, anaerobic, cellulolytic thermophile that produces one of the most efficient enzymatic systems to digest cellulose [1]. The cellulolytic capacities of *C*. *thermocellum* have been the subject of study for many years [2], and the main motivation in these efforts has been the production of high-value products, such as ethanol, from cellulosic wastes [3]. To solubilize such carbohydrates, *C*. *thermocellum* secretes a multi-enzyme complex termed the cellulosome that is anchored to the cell surface [4,5]. Although during the exponential phase of growth most of the cellulosomes are cell-associated, part of them are released from the cells into the milieu [4,6,7].

The *C*. *thermocellum* cellulosome consists of a nonhydrolytic scaffoldin subunit CipA that integrates various catalytic subunits into the complex [8,9]. Depending on the *C*. *thermocellum* strain, the scaffoldin can attach 8 or 9 catalytic subunits; e.g., the CipA scaffoldin of strain DSM 1313 attaches 8 catalytic subunits, whereas that of ATCC 27405 attaches 9 catalytic subunits [10]. Additionally, the scaffoldin subunit has a family 3 carbohydrate-binding module (CBM3) that binds the cellulosome to cellulose [8,11]. *C*. *thermocellum* can express over 80 different cellulosomal components encoded in its genome, which include an arsenal of different saccharolytic enzymes, such as, cellulases, hemicellulases, pectin-degrading enzymes and a chitinase [12,13]. This battery of enzymes helps *C*. *thermocellum* to unwrap its preferred substrate, cellulose, that is covered with different types of polysaccharides in the plant cell wall [5]. During the saccharolytic process, the enzymatic content of the cellulosome is adjusted to suit the type of polysaccharide present in the biomass [14–16]. Hence, *C*. *thermocellum* should possess biomass-sensing mechanisms that allow the cells to detect which polysaccharide(s) is(are) present in the environment and regulate the relevant genes accordingly the enzymatic requirements. At present, however, the regulation of cellulosomal genes is poorly understood.

During the course of our efforts to gain knowledge about the biomass-sensing mechanisms in *C*. *thermocellum*, our research group discovered a collection of eight alternative σ factors and their cognate membrane-associated anti-σ factors that may play a role in regulating genes encoding cellulosomal enzymes and other proteins [17]. In the *C*. *thermocellum* genome, these alternative σ factor genes are positioned adjacent to their anti-σ factor genes in an operon-like organization [17]. This set of eight alternative σ factors (*C*. *thermocellum* σ^I1^ to σ^I8^) are related to the *B*. *subtilis* σ^I^ [17,18], and the expression of six of them (σ^I1^ to σ^I6^) was shown to be influenced by the presence of polysaccharides (e.g., cellulose and xylan) in the growth medium [19]. Furthermore, a recent study performed by Wei and colleagues [16] showed that *C*. *thermocellum sigI3-rsgI3*, *sigI4-rsgI4* and *sigI7-rsgI7* operons are up-regulated when the bacterium was grown in dilute acid-pretreated yellow poplar. Additionally, *in vitro* experiments showed that σ^I1^ directed the transcription from *sigI1* promoter and from the promoter of the gene *cel48S* [19] that encodes for the most abundant cellulosomal enzyme Cel48S [12,20].

The *C*. *thermocellum* anti-σ^I^ factors of σ^I1^ to σ^I6^ (RsgI1 to RsgI6) embody three domains: (I) a C-terminal carbohydrate-binding module (CBM) localized on the outer cell surface, (II) an internal transmembrane/wall-spanning segment, and (III) an N-terminal cytoplasmic portion (RsgI-N) which would bind the cognate σ^I^ factor [17,18]. The N-terminal segments (~165 residues) of the *C*. *thermocellum* RsgI proteins resemble *B*. *subtilis* RsgI, a negative regulator of its cognate σ^I^ factor [17,18]. Moreover, the binding capacities of the N-terminal cytoplasmic portions of RsgI1, RsgI2 and RsgI6 to their corresponding σ^I^ factors was demonstrated *in vitro* [19].

The C-terminal domains of the RsgIs showed binding capacities to different polysaccharides, including cellulose (RsgI1, RsgI2, RsgI4 and RsgI6), xylan (RsgI6), and pectin (RsgI3) [17,21,22]. Additionally, the crystal structures of the C-terminal CBMs of RsgI1, RsgI2 and RsgI4 were solved showing a high degree of similarity to the family 3 CBMs [22]. In the case of RsgI3, its C-terminal CBM is constituted by two tandem PA14-superfamily motifs (pfam07691, smart00758) that are found in a wide variety of other bacterial and eukaryotic proteins, which include the anthrax protective antigen (PA) [23], and the PA14 modular dyad was predicted to be a putative CBM by virtue of its binding to pectin-like polysaccharides [17]. Interestingly, the C-terminal domain of RsgI6 belongs to the glycoside hydrolase family 10 (GH10), however, its catalytic activity was shown to be very low [17,21]. Nevertheless, RsgI6-GH10 retains its binding capacity to its corresponding carbohydrates, suggesting an evolutionary adaptation to function as a polysaccharide-binding domain rather than an authentic enzymatic component [21].

The multiple *C*. *thermocellum* alternative σ^I^ factors resemble to some extent the ECF (**e**xtra**c**ytoplasmic **f**unction) σ factors [24–26], since they share common characteristics which include the following: (I) both kinds of σ factors autoregulate their own expression; (II) both kinds of σ factors are usually co-transcribed with another ORF encoding a transmembrane anti-σ factor that controls the activity of its cognate σ factor; (III) the anti-σ factor is composed of an extracytoplasmic sensory domain and an intracellular inhibitory domain that binds the σ factor; (IV) the activity of the σ factor is induced by inhibiting activity of the anti-σ factor [18,25]. We assume that the main difference between σ^I^-like factors and ECF σ factors is related to their "architectures" (Muñoz-Gutierrez et al, unpublished). While the ECF σ factors are formed with only two of the four domains of the σ^70^ family of proteins (σ_2_ and σ_4_) [25,26], the σ^I^-like factors have only one predictable functional domain associated with the amino-terminal sequence, σ_2_, and the sigma domain σ_4_ is absent [17]. In lieu of the sigma domain σ_4_, the σ^I^ factors contain a novel 100-residue conserved C-terminal domain termed σ_I-C_ [17], that might serve to recognize -35 sequences of the σ^I^ promoters.

Until now, the knowledge we have regarding the regulation of cellulosomal genes by *C*. *thermocellum* σ^I^-like factors is a recent report of Sand and co-workers [27] which showed that the xylanase genes *xyn10Z* (or Clo1313_2635 according to the DSM 1313 genome annotation), *xyn11B* (Clo1313_0522) and *xyn10D* (Clo1313_0177) were under the control of σ^I6^. Previously, Nataf and co-workers [19] showed that the cellulase gene *celS* (*cel48S*) was likely under the control of σ^I1^. Therefore, the present work was devoted to demonstrating whether individual *C*. *thermocellum* σ^I^ factors regulate specific cellulosomal target genes. Taking advantage of the fact that the transcription start sites of *C*. *thermocellum sigI6*, *xyn10Z* and *xyn11B* were mapped previously in our research group [19,27], we performed a bioinformatics analysis to identify σ^I^-dependent promoters in the genome of *C*. *thermocellum* DSM 1313. This analysis allowed us to identify 40 possible σ^I^-dependent promoters upstream of the *sigI*-like genes and certain cellulosomal genes of *C*. *thermocellum*. To corroborate the functionality of the 40 predicted promoters, we fused their DNA sequences to a promoterless *lacZ* reporter gene. To overcome the lack of genetic tools in *C*. *thermocellum*, we used a *B*. *subtilis* Δ(*sigI*-*rsgI*) strain as a heterologous host and studied the activation of the 40 predicted promoters by *C*. *thermocellum* σ^I6^ and σ^I3^. This strategy allowed us to show that *C*. *thermocellum* σ^I6^ could recognize the predicted promoters associated with *sigI6*, *sigI1*, *rsgI5*, *xyn11B*, *xyn10D*, *xyn10Z*, *xyn10Y*, *cel9V*, *cseP* and the major scaffoldin *cipA*. Additionally, *C*. *thermocellum* σ^I3^ could recognize the predicted promoters detected upstream of *sigI3*, *pl11* (Clo1313_1983 encodes a family 11 polysaccharide lyase (PL11) containing a CBM35 and a dockerin), *ce12* (Clo1313_0693 encodes for a protein that contains two family 12 carbohydrate esterase (CE12), a CBM35 and a dockerin) and *cipA*. The combination of these methodologies revealed a putative *C*. *thermocellum* σ^I6^ and σ^I3^ promoter consensus. Our results show that *C*. *thermocellum* σ^I6^ and σ^I3^ factors expressed in *B*. *subtilis* can recognize its potential promoters, supporting our hypothesis that the multiple *C*. *thermocellum* σ^I^–like factors might regulate cellulosomal genes.

## Material and Methods

### Bacterial strains, growth media and culture conditions

*C*. *thermocellum* strain DSM 1313 (LQ8) was obtained from the DSMZ (German Collection of Microorganisms and Cell Cultures, Braunschweig, Germany). The *B*. *subtilis* strains constructed in this work are isogenic derivatives of the *B*. *subtilis* strain PY79 (laboratory stock) [28]. Additional information regarding all derivatives of *B*. *subtilis* PY79 that were constructed in this work is shown in S3 Table. *B*. *subtilis* BKE13460 was obtained from the BGSC (Bacillus Genetic Stock Center, Ohio, USA). *Escherichia coli* DH5α (BioSuper Competent Cells, Bio-Lab Ltd, Jerusalem, Israel) was used for plasmid propagation during plasmid construction.

*E*. *coli* and *B*. *subtilis* were grown routinely at 37°C in liquid (at 250 rpm) or on solid LB-agar Broth (Lennox, Difco, BD Diagnostics, Maryland, USA). During β-galactosidase activity assays, *B*. *subtilis* was grown in Spizizen´s minimal medium (SMM) employing 5 g/L fructose as carbon source and supplemented with trace elements. The SMM contained (per liter) 2 g (NH_4_)_2_SO_4_, 14 g K_2_HPO_4_, 6 g KH_2_PO_4_, 1 g Na_3_Citrate·2H_2_O, and 0.2 g MgSO_4_·7H_2_O. The trace elements used were (per liter) 125 mg MgCl_2_·6H_2_O, 5.5 mg CaCl_2_, 13.5 mg FeCl_2·_6H_2_O, 1 mg MnCl_2_·4H_2_O, 1.7 mg ZnCl_2_, 0.43 mg CuCl_2_·2H_2_O, 0.6 mg CoCl_2_·6H_2_O, and 0.6 mg Na_2_MoO_4_·2H_2_O. When appropriate, antibiotics were included at the following final concentrations: 100 μg/mL ampicillin (Amp), 50 μg/mL kanamycin (Kan), 100 μg/mL spectinomycin (Spt), 5 μg/mL chloramphenicol (Cam) or 3 μg/mL erythromycin (Erm). The induction of genes under the P_*xylA*_ promoter was carried out with D-xylose (10 g/L final). All chemicals were purchased from Sigma-Aldrich (Missouri, USA).

### DNA manipulation techniques

The oligonucleotide primers used in the present study are shown in S1 Table. Standard procedures were employed for DNA isolation, polymerase chain reaction (PCR), restriction-enzyme digestion, dephosphorylation, transformations, and gel electrophoresis as described elsewhere [29]. Plasmids were built using a combination of standard molecular cloning techniques [29] and ligase-independent cloning using the In-Fusion HD Cloning Kit (Clontech Laboratories, Inc., California, USA). *C*. *thermocellum* DNA sequences were PCR-amplified using *C*. *thermocellum* DSM 1313 genomic DNA as template. The upstream and downstream regions of the *B*. *subtilis sigI-rsgI* operon were PCR-amplified using *B*. *subtilis* PY79 genomic DNA as template. The *lox71*-*erm*-*lox66* cassette was PCR-amplified using *B*. *subtilis* BKE13460 genomic DNA as template. Amplification of DNA for cloning was performed using TaKaRa Ex Taq (Takara Bio Inc., Shiga, Japan). Colony PCR was performed using Hy-Taq Ready Mix (Hy Laboratories Ltd, Rehovot, Israel). PCR primers were purchased from hy·labs (Hy Laboratories Ltd). Restriction enzymes, alkaline phosphatase, and ligase were purchased from Fermentas (Thermo Fisher Scientific Inc., Massachusetts, USA). PCR and agarose-gel products were isolated and purified using the hy·labs Gel/PCR Extraction Kit (Hy Laboratories Ltd). Purification of plasmids was carried out using the Presto™ Mini Plasmid Kit (Geneaid Biotech Ltd., Shijr, Taiwan). All clones were verified by PCR and sequencing in the Instrumentation and Service Center of the Life Sciences Faculty at Tel Aviv University.

### Construction of plasmids

Plasmids constructed in the present work are listed in S2 Table. The pLOXErysigIrsgIBs plasmid was constructed to knockout the *B*. *subtilis sigI-rsgI* operon including its promoter using resistance to Erm as a selective marker. The upstream (464 bp) and downstream (505 bp) regions of the *B*. *subtilis sigI-rsgI* operon were PCR-amplified using primer pairs P1-P2 and P3-P4, respectively. The *lox71*-*erm*-*lox66* cassette was amplified using primer pair P5-P6. Subsequently, the three PCR products were cloned simultaneously with the In-Fusion HD Cloning Kit into a linearized pUC19 vector (generated by PCR and provided with the kit) following the kit protocol, obtaining the pLOXErysigIrsgIBs plasmid (S1 Fig).

To express the *C*. *thermocellum* σ^I6^ and σ^I3^ factors in *B*. *subtilis*, we used the pAX01 plasmid [30]. This vector was designed for integration at the *B*. *subtilis lacA* chromosomal locus, carries an *erm* resistance cassette as a selectable marker, and has the xylose-inducible promoter P_*xylA*_. First, pAX01 was linearized with the restriction enzyme BamHI. Subsequently, the DNA sequence of *C*. *thermocellum sigI6* and *sigI3* were PCR-amplified using primer pair P7-P8 and P9-P10, respectively (S1 Table). Finally, the PCR products were cloned using the In-Fusion HD Cloning Kit into the linearized pAX01 vector, obtaining the pAX01-sigI6 and pAX01-sigI3 plasmids.

To study the promoters that are under the control of *C*. *thermocellum* σ^I6^ and σ^I3^, we used the pBS1C*lacZ* plasmid that contains a promoterless *lacZ* reporter gene [31]. This vector was designed to integrate at the *B*. *subtilis amyE* locus and carries a *cat*-resistance cassette as a selectable marker. The upstream region of the *C*. *thermocellum* σ^I^-factor genes that contain the predicted promoter and the upstream region of some cellulosomal genes that contain predicted σ^I^-dependent promoters were PCR-amplified, using the primer pairs listed in S1 Table (primers P11 to P90). Subsequently, each PCR product was digested with restriction enzymes EcoRI and BamHI. Finally, each digested PCR was cloned into the pBS1C*lacZ* plasmid that was digested previously with the same restriction enzymes, thus obtaining the pBS1C*lacZ* derived plasmids listed in S2 Table.

In order to study the important bases for promoter recognition by *C*. *thermocellum* σ^I6^, mutant versions of the *xyn10Z* σ^I6^-dependent promoter were created by site-directed mutagenesis. To introduce mutations in the conserved bases of the -35 element, the forward primers from P93 to P99, which contain the mutated nucleotides, were used with the reverse primer P92 (S1 Table). To introduce individual mutations in the conserved bases of the -10 element, the reverse primers from P100 to P105, which contain the mutated nucleotides, were used with the forward primer P91 (S1 Table). In order to compare the mutant version, a short version of the *xyn10Z* σ^I6^-dependent promoter with the same length of the mutant versions was PCR-amplified using the primer pair P91-P92 (S1 Table). Subsequently, each PCR product was digested with restriction enzymes EcoRI and BamHI. Finally, each digested PCR was cloned into the pBS1C*lacZ* plasmid that was digested previously with the same restriction enzymes, thereby obtaining the pBS1C*lacZ*-derived plasmids listed in S2 Table.

### Construction of *B*. *subtilis* strains

*B*. *subtilis* was transformed by using the natural competence method [32]. Chromosomal integration of plasmids by a double-crossover event was confirmed by colony PCR using the primer pairs listed in S1 Table (primers P106 to P115). The different *B*. *subtilis* strains obtained were stored at -80°C in 20% (v/v) glycerol. The strains constructed in the present work are listed in S3 Table.

To construct a *B*. *subtilis* PY79 devoid of its *sigI-rsgI* operon, *B*. *subtilis* PY79 was first transformed with the pLOXErysigIrsgIBs plasmid, and the cells were selected with Erm, obtaining the *B*. *subtilis* CO01 strain. Subsequently, *B*. *subtilis* CO01 was transformed with the pDR244 plasmid (obtained from the BGSC) that encodes the sequence of the Cre recombinase and has a thermosensitive origin of replication. The cells were plated on LB-agar containing Spt and were incubated at 30°C. Several individual colonies were then streaked on a plain LB-agar plate and incubated overnight at 42°C to cure pDR244. The resulting colonies were screening for plasmid curing (Spt sensitivity) and the loss of the *lox71*-*erm*-*lox66* cassette (Erm sensitivity). Finally, a single colony was streaked on plain LB-agar plate and grown at 37°C. The loss of the *lox71*-*erm*-*lox66* cassette was confirmed by PCR with the primer pair P118-P119 (S1 Table) thus obtaining the *B*. *subtilis* CO02 strain (S3 Table).

### β-Galactosidase activity assays

To measure the β-galactosidase activity, strain samples were taken from the -80°C glycerol stock and inoculated in 5 mL of SMM with Cam. Subsequently, the cells were grown overnight at 37°C with shaking (250 rpm). The next day, the cells were inoculated in 2.5 mL of SMM to an OD_600_ between 0.1–0.2 and grown at 37°C (250 rpm). When the cells reached mid-log growth phase (approx. 0.4–0.5 OD_600_), the culture was separated into two tubes, and one tube was supplemented with xylose (1% final concentration) whereas the other was used as a blank. Then, the cells were allowed to grow for another hour at 37°C (250 rpm). Finally, the cells were recovered by centrifuging at 16,000 g for 5 min, washed twice with Z-buffer (60 mM Na_2_HPO_4_·7H_2_O, 40 mM NaH_2_PO_4_, 10 mM KCl, 1 mM MgSO_4_·7H_2_O, and 50 mM β-mercaptoethanol, pH 7.0) and recovered in 0.5 mL of Z-buffer.

Enzymatic activity was measured with the fluorogenic substrate (4 mg/mL) 4-methylumbelliferyl β-D-galactopyranoside (4-MUG, Sigma-Aldrich) in a microplate reader (Biotek Synergy HT, Vermont, USA). The cells, recovered in Z-buffer (150 μL), were placed in a 96-well plate and 2 mL of MUG (4 mg/mL) were added to initiate the enzymatic reaction. The release of the fluorescent compound 4-methylumbelliferone (4-MU) was measured (using the excitation filter 360/40 and the emission filter 460/40) every 10 min at 30°C with medium agitation for one hour. The reaction was stopped by adding 100 μL of 1M Na_2_CO_3_. To calculate the β-galactosidase activity, a standard curve with 4-MU was prepared. One unit of enzyme activity was defined as the amount of β-galactosidase that releases 1 μmol of 4-MU per minute. All the β-galactosidase activities were normalized with cell density (OD_600_).

### Bioinformatics analysis

Primary DNA sequence analyses and DNA motif searches were performed using the Clone Manager 9 Professional Edition software (Scientific & Educational Software, Durham, NC). The *B*. *subtilis* 168 and *C*. *thermocellum* DSM 1313 *sigI* genes and their promoter sequences (extracted from GenBank NZ_CP010052.1 and NC_017304.1, respectively) were used as BLAST [33] queries to mine public databases including that at the National Center for Biotechnology Information (NCBI) (http://www.ncbi.nlm.nih.gov/). In order to prevent a possible loss of promoter candidates during BLAST mining, we used both discontiguous megablast ("more dissimilar sequences") as well as blastn ("somewhat similar sequences") as implemented at NCBI (http://blast.ncbi.nlm.nih.gov/Blast.cgi). Pairwise and multiple sequence alignments were performed with the CLUSTALW program [34] using either the Network Protein Sequence Analysis server (http://npsa-pbil.ibcp.fr/cgi-bin/npsa_automat.pl?page=/NPSA/npsa_clustalw.html), or the ClustalW2 at the EMBL-EBI (http://www.ebi.ac.uk/Tools/msa/clustalw2/). WebLogos [35] were generated by using a public logo generator web application (http://weblogo.berkeley.edu/).

## Results

### Bioinformatics comparison of promoter sequences of alternative σ^I^ factor from various cellulosome-producing bacteria and *Bacillales* species

**Fig 1:**
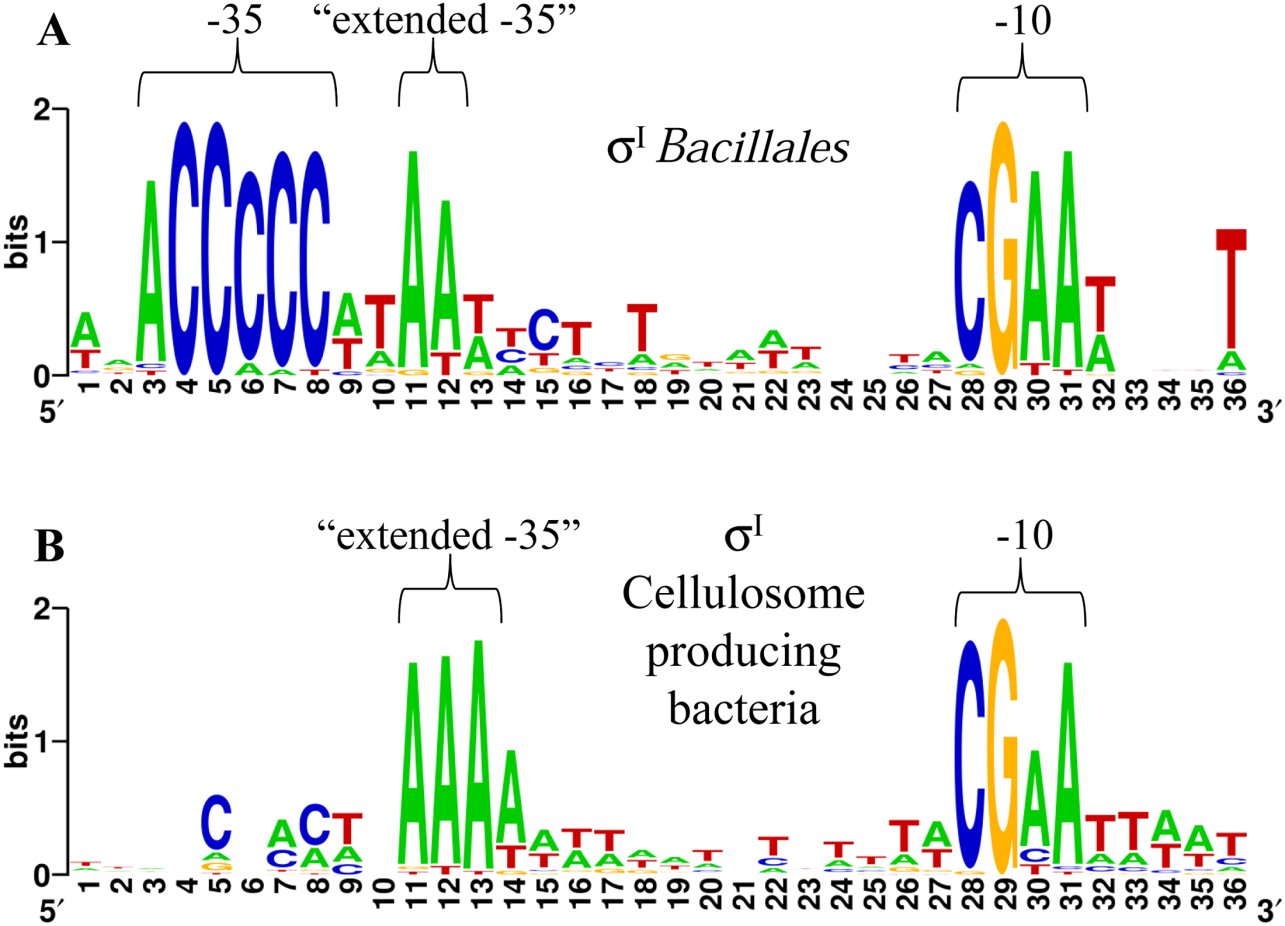
Identification of conserved elements of σ^I^-dependent promoter sequences. (A) WebLogo generated with the *Bacillales sigI* promoters shown in S4 Table. (B) WebLogo generated with the *C*. *thermocellum* and *C*. *straminisolvens sigI* promoters shown in Table 1, and the *C*. *clariflavum*, *A*. *cellulolyticus and Pseudobacteroides cellulosolvens sigI* promoters shown in S5 Table.

In order to identify the conserved sequence motifs that could be used for the analysis of putative *C*. *thermocellum sigI*-like gene promoters, we performed multiple sequence alignments of the experimentally detected σ^I^-dependent promoter sequences. The initial analysis was performed using the *sigI* promoter sequences experimentally identified in *B*. *subtilis*, *Bacillus licheniformis* ATCC 14580, *Bacillus thuringiensis* serovar israelensis ATCC 35646 and *Bacillus* sp. strain NRRL B-14911 [18,36]. Additionally, the experimentally identified promoters of the σ^I^-dependent genes *bcrC* and *mreBH*, which are involved in cell envelope integrity and homeostasis during heat stress in *B*. *subtilis* [36], were also included. The analysis was improved by including DNA sequences located immediately upstream of *sigI*-like genes in various species of the order *Bacillales*. The alignment is shown in S4 Table, and a high conservation of two short DNA sequences upstream of the *Bacillales sigI*-*rsgI* operons can be observed. These basic putative promoter motifs can also be observed in Fig 1A that shows a WebLogo generated with the *Bacillales* σ^I^-dependent promoters shown in S4 Table. As already proposed by Tseng and Shaw [36], the suggested *Bacillales* σ^I^ promoter consensus sequence is ACCCCC for the -35 element and CGAA for the -10 element (Fig 1A and S4 Table). Interestingly, a conserved sequence AA downstream of the -35 element (already mentioned by Tseng and Shaw [36]), and a conserved T downstream of the -10 element can also be observed (Fig 1A and S4 Table). For future comparisons, we named the conserved sequence AA as "extended -35".

The deduced consensus sequences of the *Bacillales sigI* promoter elements were used to find sequence similarities between the predicted promoters of the different *C*. *thermocellum sigI* genes. The upstream intergenic regions of the eight *C*. *thermocellum sigI* genes were manually analyzed, focusing on potential conservation of the consensus sequences of the *Bacillales sigI* promoter elements -35 and -10. Selected promoter candidate sequences were then used for multiple sequence alignments using the ClustalW algorithm [34]. The analysis was facilitated by the fact that the transcription start sites of *C*. *thermocellum sigI1* and *sigI6* genes were previously identified by Nataf and co-workers [19]. Additionally, the analysis was improved by comparing the *C*. *thermocellum* predicted promoter sequences to those of another cellulosome-producing species, *C*. *straminisolvens*, whose genome (NCBI Reference Sequence: NZ_BAVR00000000.1) has a very high similarity to that of *C*. *thermocellum* (96.2% similar based on 16S rDNA) [37,38]. The multiple promoter sequence alignment is presented in Table 1. The putative promoters of *C*. *thermocellum sigI2*, *sigI3*, *sigI4*, *sigI7*, *sigI8*, and those of *C*. *straminisolvens sigI1*, *sigI2*, *sigI3*, *sigI4*, *sigI6*, *sigI7* and *sigI8* were predicted. As shown in Table 1, a conserved AAA sequence for the "extended -35" element and a highly conserved CGWA for the -10 element were identified. Moreover, a highly conserved C upstream of the "extended -35" was also identified (Table 1).

**Table 1:** Alignment of putative *C*. *thermocellum* and *C*. *straminisolvens sigI* promoters.

Gene^a^	Locus tags^b^	RsgI-C terminal domain	Promoter region 5'→3'	5' UTR
*Ct sigI1-rsgI1*	Clo1313_2174–2173, Cthe_0058–0059	CBM3	taatatacacaa**AAA**aa-gcagatgtata**CGa****A**gtaatctact***G***	16
*Cs sigI1-rsgI1*	JCM21531_2023–2024	CBM3	taatatacataa**AAA**aa-gcaggcttgaa**CGaA**gtaatctactg	17
*Ct sigI2-rsgI2*	Clo1313_1961–1962, Cthe_0268–0267	CBM3	tggtat**C**ccccg**AAA**aaatgttcccttta**CGaA**ataactagtaa	147
*Cs sigI2-rsgI2*	JCM21531_2790–2789	CBM3	tgatat**C**cccct**AAA**atttgttcctctta**CGaA**ataacttatta	159
*Ct sigI3-rsgI3*	Clo1313_1911–1910, Cthe_0315–0316	2xPA14	tatgaa**C**ccctc**AAA**aaaatcatttggtg**CGtA**caagtattgaa	13
*Cs sigI3-rsgI3*	JCM21531_2365–2366	2xPA14	tgtaaa**C**ccctc**AAA**aaa-taactttgtg**CGtA**caagtattaaa	15
*Ct sigI4-rsgI4*	Clo1313_1818–1817, Cthe_0403–0404	CBM3	aacgtc**C**agctg**AAA**attttctgccacgc**CG**c**A**ttaattttttt	13
*Cs sigI4-rsgI4*	JCM21531_1812–1813	CBM3	aacgtc**C**aacta**AAA**gtttgttgccacat**CG**c**A**ttaatctattt	13
*Ct sigI6-rsgI6*	Clo1313_2778–2777, Cthe_2120–2119	GH10	acaatg**C**gacat**AAA**accattccggtata**CGaA**tcgatataa***G***a	20
*Cs sigI6-rsgI6*	JCM21531_4109–4108	GH10	atgatg**C**gacat**AAA**gctattccagtcta**CGaA**ttcatatagga	22
*Ct sigI7-rsgI7*	Clo1313_0104–0105, Cthe_2521–2522	UNK	attcga**C**tgatgtt**A**tt-taaatttgtgt**CGaA**ctttgctgatg	52
*Cs sigI7-rsgI7*	JCM21531_3721–3720	UNK	attcga**C**tagtggtttg-tagatttatgt**CGaA**ctttgctgaca	61
*Ct sigI8-rsgI8*	Clo1313_0525–0524, Cthe_2975–2974	UNK	actttc**C**gaatc**AAA**atgaaatccatata**CGaA**ttttctatagt	16
*Cs sigI8-rsgI8*	JCM21531_4043–4045	UNK	ttttac**C**gaatt**AAA**atagaagtcatata**CGaA**tcctctatagc	18
Consensus			**C**-----**AAA 13-14(N) CGWA**	
*Bacillales* consensus			**ACCCCC**--**AA 15(N) CGAA**----**T**	

The most conserved bases (> 85%) are shown in bold capital fonts. Transcription start sites in *Ct sigI1-rsgI1* and *Ct sigI6-rsgI6*, identified by Nataf and co-workers [19], are shown in uppercase bold italics; and the promoter regions proposed by the same authors are underlined [19]. *Ct*, *Clostridium thermocellum*; *Cs*, *Clostridium straminisolvens*. W represents A or T.

^*a*^ *C*. *thermocellum* and *C*. *straminisolvens sigI5* are not included in the table. During the analysis we failed to predict a σ^I^-dependent promoter sequence for both *C*. *thermocellum* and *C*. *straminisolvens sigI5*, because of the low similarity of the upstream intergenic regions of these genes.

^*b*^ Clo1313 and Cthe are the locus tag prefixes of *C*. *thermocellum* strains DSM 1313 and ATCC 27405, respectively.

It is important to mention that during this analysis we failed to predict a σ^I^-dependent promoter sequence for both *C*. *thermocellum* and *C*. *straminisolvens sigI5* genes, owing to the low similarity of their upstream intergenic regions. Interestingly, whereas most of the sequences of the *rsgI* genes overlap with sequences of their cognate *sigI* genes, *sigI5* and *rsgI5* genes are separated by an intergenic region of 97 nucleotides, which contains a predicted σ^I^-dependent promoter (Table 2). This suggests a different type of gene organization and regulation of *sigI5* and *rsgI5* in both *C*. *thermocellum* and *C*. *straminisolvens*.

**Table 2:** Alignment of predicted *C*. *thermocellum* σ^I^-dependent promoters.

Gene^a^	Locus Tags^b^	Promoter region 5'→3'	5' UTR
*xyn10Z* ^c^	Clo1313_2635, Cthe_1963	accgacac**AAA**aatgtgagcgttca**CGaA**acaa**Ta**aat***A***t	96
*xyn11B* ^c^,^d^	Clo1313_0522, Cthe_2972	agcgactt**AAA**aaattatatttttg**CGaA**taga**Ta**ata***T***g	164
*ce8*	Clo1313_0500, Cthe_2949	ccccgctc**AAA**tgttgcataaacct**CGaA**tctta**a**aataa	32
*ce12*	Clo1313_0693, Cthe_3141	taccctta**AAA**aaacttgct-tctaCGtAtttta**a**tatta	51
*cel5E*	Clo1313_1425, Cthe_0797	gctgtcca**AAA**gaaaa-tgattttt**CGaA**ttaa**Ta**taata	156
*cel8A*	Clo1313_1960, Cthe_0269	accctatc**AAA**taacccattcaattCGcAttta**Tt**ttaag	254
*cel9J*	Clo1313_1604, Cthe_0624	gccccctt**AAA**aaatttta-aaatt**CGaA**attaa**t**ttttg	477
*cel9P*	Clo1313_1955, Cthe_0274	aacgtctat**AA**tttttt-atgataaCGataaaa**Tt**aaatt	19
*cel9Q*	Clo1313_1603, Cthe_0625	acccactt**AAA**aatgtgtatgtgcaCGgAtttc**Ta**tttgg	375
*cel9U*^e^	Clo1313_3023, Cthe_2360	agcccctc**AAA**aattttttcccttt**CGaA**tata**Ta**tagat	394
*cel9V*	Clo1313_0349, Cthe_2760	atacccat**AAA**atttttatgttcta**CGaA**tata**Ta**atata	124
*cel48S*	Clo1313_2747, Cthe_2089	gccccctc**AAA**aagtatattttttt**CGaA**gata**Ta**tatat	498
*cenC*	Clo1313_0420, Cthe_2879	cccaatcg**AAA**aaagaacatgtcat**CGaA**tcta**Ta**tatca	102
*cipA*	Clo1313_0627, Cthe_3077	tgcccctc**AAA**ttccgtttatatat**CGaA**tata**Ta**ttaca	846
*cseP*	Clo1313_2188, Cthe_0044	taagccac**AAA**attattt-tttcta**CGaA**tata**Ta**ttgaa	132
*pelB2*	Clo1313_0501, Cthe_2950	tcccaatg**AAA**tacgacccttgataCGtAttat**Ta**atata	67
*pilZ*	Clo1313_1490, Cthe_0733	gccccctc**AAA**ata-tgagaacatt**CGaA**atat**Ta**taata	321
*pl11*	Clo1313_1983, Cthe_0246	ctacccct**AAA**aaaa-ttagaatttCGtAttta**Ta**aaaag	39
*rsgI5*	Clo1313_0985, Cthe_1273	atggacca**AAA**agtactttcaaaca**CGaA**atta**Tt**aaata	43
*rsgI9*	Clo1313_1969, Cthe_0260	ctctaaaaAtAtcgggatttttttc**CGaA**ataac**t**aatag	31
*sdbA*	Clo1313_0950, Cthe_1307	caacgctcAAtacgaactctttctc**CGaA**ttta**Tt**ctatt	157
*xgh74A*	Clo1313_0851, Cthe_1398	ggtacatc**AAA**ggaaagtacaggtc**CGaA**ttta**Ta**tagcg	147
*xyn10D*	Clo1313_0177, Cthe_2590	tgcgacca**AAA**ggcgtcaaatttca**CGaA**ataca**t**ataaa	33
*xyn10Y*	Clo1313_1305, Cthe_0912	cccaacgt**AAA**aattcaataccttt**CGaA**taaa**Ta**acata	277
GH30-CBM6-Doc	Clo1313_0563, Cthe_3012	ccgtacat**AAA**aagaagttttgatt**CGaA**taat**Ta**acaca	67
GH43-2xCBM6-Doc	Clo1313_0987, Cthe_1271	cccaaccc**AAA**cttgccatatgtttCGtAcaaa**Ta**aattg	67
HP	Clo1313_1436, Cthe_0785	atcccctttAAgaattgacataaaaCGcAttaac**t**attat	106
HP-Doc	Clo1313_1494, Cthe_0729	acggaaat**AAA**aacaactccaatta**CGaA**taaa**Ta**tacca	35
GH43-CBM42-Doc	Clo1313_2216, Cthe_0015	cccactcc**AAA**aaacatttaattctCGtAttat**Ta**taaca	46
GH39-2xCBM35-Doc	Clo1313_2793, Cthe_2137^f^	ctcaactt**AAA**aaatacattcttctCGtAtatg**Ta**agtta	160
GH43-CBM42-Doc	Clo1313_2794, Cthe_2138	tacgtcac**AAA**ccaaaaacccagaa**CGaA**ccaa**Tt**aataa	121
GH2-CBM6-Doc	Clo1313_2861, Cthe_2197^f^	cccaacta**AAA**aaaataggtacttcCGtAaaag**Ta**aaaca	163
ABC transporter	Clo1313_2866^f^	taacccta**AAA**atttaatgccgatt**CGaA**taaaa**a**agcct	149
Consensus		**AAA 13-14(N) CGWA----TW**	
*Bacillales* consensus		**ACCCCC--AA 15(N) CGAA----T**	

The most conserved bases (> 85%) are shown in bold capital fonts. W represents A or T.

^*a*^ The genes without trivial names are denoted with their main protein product domain(s). GH, glycoside hydrolase; CBM, carbohydrate-binding module; Doc, dockerin. HP, hypothetical protein.

^*b*^ Clo1313 and Cthe are the locus tag prefixes of *C*. *thermocellum* strains DSM 1313 and ATCC 27405, respectively.

^*c*^ Transcription start sites identified by Sand and co-workers [27] are indicated in uppercase bold italics, and the underlined sequences are the promoter sequence proposed by the same authors [27].

^*d*^ In *C*. *thermocellum* DSM 1313 *xynB* is part of the operon *xynB-xynA*, whereas *xynB* is absent in other strains, such as ATCC 27405 and JW20.

^*e*^ All the promoter sequences shown in the table are exactly the same for both *C*. *thermocellum* strains, DSM 1313 and ATCC 27405. The only small difference is in the gene *cel9U* at the 5' position. Whereas strain DSM 1313 has the sequence 5' AGCCCCTC**AAA** 3' (bold fonts are part of the consensus in the -35 element), the sequence of strain ATCC 27405 is 5' AGCTCCCTC**AAA** 3' (underline designates an inserted T).

^*f*^ In *C*. *thermocellum* strain ATCC 27405 the genes Cthe_2137 and Cthe_2197 are interrupted by an IS element. Additionally, strain ATCC 27405 does not contain a gene orthologous to Clo1313_2866 (an ABC transporter ATP-binding protein).

To investigate how conserved are the "extended -35" and -10 elements of *C*. *thermocellum* and *C*. *straminisolvens sigI*-like gene promoters, we performed a search of *sigI-rsgI* operons using the publicly available genomic sequences of the known cellulosome-producing bacteria. The S5 Table shows the putative promoters upstream of *sigI*-like genes found during the mining. Multiple *sigI*-like genes in *Clostridium clariflavum*, *Acetivibrio cellulolyticus* and *Pseudobacteroides cellulosolvens* were found, and most of their cognate *rsgI*-like genes encode proteins containing a C-terminal CBM. As shown in S5 Table, a conserved AAA sequence for the "extended -35" element and a highly conserved CGWA for the -10 element were identified in *C*. *clariflavum*, *A*. *cellulolyticus* and *P*. *cellulosolvens*. These results confirm the high conservation of the -35 and -10 elements in *sigI*-like promoters of taxonomically divergent cellulosome-producing bacteria. The high conservation of the "extended -35" and -10 promoter elements of cellulosome-producing bacteria is more evident in the WebLogo generated with the predicted promoter sequences of *C*. *thermocellum*, *C*. *straminisolvens*, *C*. *clariflavum*, *A*. *cellulolyticus* and *P*. *cellulosolvens* shown in Fig 1B.

Comparison between the predicted promoters of the *sigI*-like genes of cellulosome-producing bacteria and the promoter consensus sequence of *Bacillales sigI* genes shows a different level of similarities (Table 1 and S5 Table). For example, while the putative promoters of *C*. *thermocellum* and *C*. *straminisolvens sigI2* and *sigI3* are most similar to the promoter consensus sequence of *Bacillales sigI* genes, the putative promoters of *C*. *thermocellum* and *C*. *straminisolvens sigI7* are less similar (Table 1). This observation is quite interesting because the deduced amino acid sequence of *C*. *thermocellum* σ^I7^ has the highest similarity to *B*. *subtilis* σ^I^ (data not shown). Our analysis shows that the cellulosome-producing bacteria which use multiple *sigI*-like genes probably maintain different levels of similarity in promoter sequences to fine-tune the regulation of individual *sigI*-like genes, as well as cellulosomal target genes. With the predicted promoter sequences of the multiple *sigI*-like genes of *C*. *thermocellum*, *C*. *straminisolvens*, *C*. *clariflavum*, *A*. *cellulolyticus* and *P*. *cellulosolvens* we suggest that AAA of the "extended -35" and CGWA of the -10 elements represent the general motifs for σ^I^-dependent promoters of cellulosome-producing bacteria.

### Searching for σ^I^–dependent promoter sequences of cellulosomal genes in *C*. *thermocellum*

Based on the assumption that σ^I^ factors autoregulate their own expression, and hence the genes that are under their control should have similar promoter sequences, we performed a search of putative promoter sequences of the cellulosomal genes of *C*. *thermocellum*. The search was performed by exploiting the conserved sequences in the "extended -35" (AAA) and -10 (CGWA) elements of the general motifs in the *sigI*-like gene promoters of cellulosome-producing bacteria. The analysis was facilitated with the recent identification of the transcriptional start sites of *C*. *thermocellum xyn10Z* and *xyn11B* by Sand and co-workers [27]. In Table 2 are listed the 33 putative predicted promoters that were identified during the analysis. Additionally, Table 2 shows the conserved AAA sequence for the "extended -35" element and the conserved CGWA for the -10 element. Interestingly, a highly conserved TW dinucleotide (W represents A or T), downstream of the -10 element, was also identified (Table 2).

### Searching for σ^I6^–dependent promoter sequences of cellulosomal genes in *C*. *thermocellum*

Given the limited genetic tool set available for *C*. *thermocellum*, we used *B*. *subtilis* as a heterologous host to test the ability of *C*. *thermocellum* σ^I6^ and σ^I3^ factors to recognize the *C*. *thermocellum* predicted promoters. A similar strategy has been successfully used by several research groups to analyze regulatory proteins from different Firmicutes species as *Clostridium difficile*, *Enterococcus faecalis* and *Oceanobacillus iheyensis* [39–42]. Additionally, the high homology presented by RNAPs of *B*. *subtilis* and *C*. *thermocellum* (e.g., more than 67% of identical residues for subunits α, β and β'; see S2 Fig) gave more support to this approach.

To avoid the interference of the native *B*. *subtilis* σ^I^ during the study of *C*. *thermocellum* σ^I^ factors, we constructed the *B*. *subtilis* CO02 strain which is devoid of its *sigI-rsgI* operon (S3 Table). The present work was first focused on the activation of putative promoter sequences by *C*. *thermocellum* σ^I6^ factor. This initial analysis was facilitated by the fact that the *C*. *thermocellum* σ^I6^ promoter was previously identified by Nataf and co-workers [19] and that the xylanase genes *xyn10Z*, *xyn11B* and *xyn10D* were shown to be under the control of σ^I6^ by Sand and co-workers [27]. The 7 predicted promoters of *C*. *thermocellum sigI*-like genes (Table 1) and the 33 *C*. *thermocellum* σ^I^-dependent predicted promoters (Table 2) were fused to a *lacZ* reporter gene (S2 Table) and integrated into the *B*. *subtilis amyE* locus (S3 Table). Subsequently, the *C*. *thermocellum* σ^I6^ factor was integrated into the *B*. *subtilis lacA* locus, and the recognition of the predicted promoters by *C*. *thermocellum* σ^I6^ was analyzed by measuring LacZ activity (Table 3). Our analysis showed that *C*. *thermocellum* σ^I6^ factor recognized 10 predicted promoters that correspond to the *C*. *thermocellum* genes *xyn10Z*, *xyn11B*, *cipA*, *sigI6*, *xyn10Y*, *cseP* (Clo1313_2188, Cthe_0044), *sigI1*, *rsgI5*, *xyn10D* and *cel9V* (Clo1313_0349, Cthe_2760). The LacZ activities of these 10 predicted σ^I6^-dependent promoters are shown in the Table 3.

**Table 3:** Quantitative evaluation of possible promoters under the control of *C*. *thermocellum* σ^I6^.

Gene	Locus tags^a^	Gene product^e^	Ref.	Activity (U)^f^
*xyn10Z*	Clo1313_2635, Cthe_1963	Cellulosomal xylanase:CE1-CBM6-Doc-GH10	[43]	1 126 ± 149
*xyn11B*^b^	Clo1313_0522, Cthe_2972^a^	Cellulosomal xylanase:GH11-CBM6-Doc	[44]	919 ± 72
*cipA*^c^	Clo1313_0627, Cthe_3077	Cellulosomal scaffoldin subunit:2(Coh)-CBM3-6(Coh)-X-Doc	[45]	266 ± 13
*cel9V*	Clo1313_0349, Cthe_2760	Cellulosomal endoglucanase:GH9-2(CBM3)-Doc	[46]	223 ± 49
*sigI6*	Clo1313_2778, Cthe_2120	Alternative σ^I6^ factor	[17]	139 ± 6
*xyn10Y*	Clo1313_1305, Cthe_0912	Cellulosomal xylanase:CBM22-GH10-CBM22-Doc-CE1	[47]	113 ± 10
*cseP*	Clo1313_2188, Cthe_0044	Cellulosomal component:CotH-Doc	[48]	93 ± 17
*sigI1*	Clo1313_2174, Cthe_0058	Alternative σ^I1^ factor	[17]	28 ± 4
*rsgI5*	Clo1313_0985, Cthe_1273	Anti-σ^I5^ factor:RsgI_N-UNK-CBM42	[17]	25 ± 5
*xyn10D*	Clo1313_0177, Cthe_2590	Cellulosomal xylanase:CBM22-GH10-Doc	[49]	20 ± 3
*sigI3*^d^	Clo1313_1911, Cthe_0315	Alternative σ^I3^ factor	[17]	ND

^*a*^ Clo1313 and Cthe are the locus tag prefixes of *C*. *thermocellum* strains DSM 1313 and ATCC 27405, respectively.

^*b*^ In *C*. *thermocellum* DSM 1313 *xyn11B* is part of the operon *xyn11B-xyn11A*, whereas *xyn11B* is absent in other strains, such as ATCC 27405 and JW20.

^*c*^ In *C*. *thermocellum* DSM 1313 *cipA* encodes for a scaffoldin with 8 cohesins, whereas in other strains, such as ATCC 27405, *cipA* encodes for a scaffoldin with 9 cohesins [10].

^*d*^ The predicted promoter of *C*. *thermocellum sigI3* was used as negative control.

^*e*^ CE, carbohydrate esterase; CBM, carbohydrate binding module; Doc, dockerin; GH, glycoside hydrolase; Coh, cohesin; X, CipA X-module; CotH, spore coat protein H; UNK, unknown domain.

^*f*^ The activity was measured using MUG as substrate. One unit of enzyme activity (U) was defined as the amount of β-galactosidase that releases 1 μmol of 4-MU per minute Numbers in parenthesis represent the standard deviation of at least three independent experiments. ND means not detected.

As expected, the heterologous expression of the *C*. *thermocellum* σ^I6^ in *B*. *subtilis* allowed the recognition of its own promoter. This result is in agreement with the recent report of Sand and co-workers [27], which shows that *C*. *thermocellum* σ^I6^ is autoregulated. Interestingly, five of the 10 activated promoters correspond to genes encoding the cellulosomal associated GH9, GH10 and GH11 glycoside hydrolases (*xyn10Z*, *xyn11B*, *xyn10Y*, *xyn10D* and *cel9V*). Furthermore, the highest β-galactosidase (LacZ) activities were obtained with the predicted promoters of the two xylanase genes *xyn10Z* and *xyn11B* (Table 3). Moreover, *xyn11B* is the first gene of the bicistronic operon *xyn11B*-*xyn11A* in *C*. *thermocellum* DSM 1313, whereas the *xyn11B* gene is lacking in other *C*. *thermocellum* strains, such as ATCC 27405 and JW20 (S6 Table). Bioinformatics analysis, performed with seven strains of *C*. *thermocellum* (DSM 1313, ATCC 27405, DSM 2360, YS, AD2, JW20 and BC1) and *C*. *straminisolvens*, showed identical predicted σ^I6^ promoter upstream of a single *xyn11A* gene of strains ATCC 27405 and JW20 (S6 Table). This suggests a strong prediction for the regulation of both xylanases, Xyn11B and Xyn11A, by the *C*. *thermocellum* σ^I6^ factor. Finally, the recognition of predicted promoters of genes encoding non-enzymatic proteins (*cipA*, *cseP*, *sigI1* and *rsgI5*) by the *C*. *thermocellum* σ^I6^ factor suggests a more complex regulon for this kind of alternative σ factors. As already mentioned, *sigI1* and *rsgI5* are also proposed to be involved in the regulation of cellulosomal genes [17]. Hence, the recognition of the *sigI1* and *rsgI5* promoters by the *C*. *thermocellum* σ^I6^ factor suggests the possibility of crosstalk between different *C*. *thermocellum* σ^I^ factors and an overlap of their respective regulons.

### Identification of conserved promoter elements for σ^I6^ recognition

**Fig 2:**
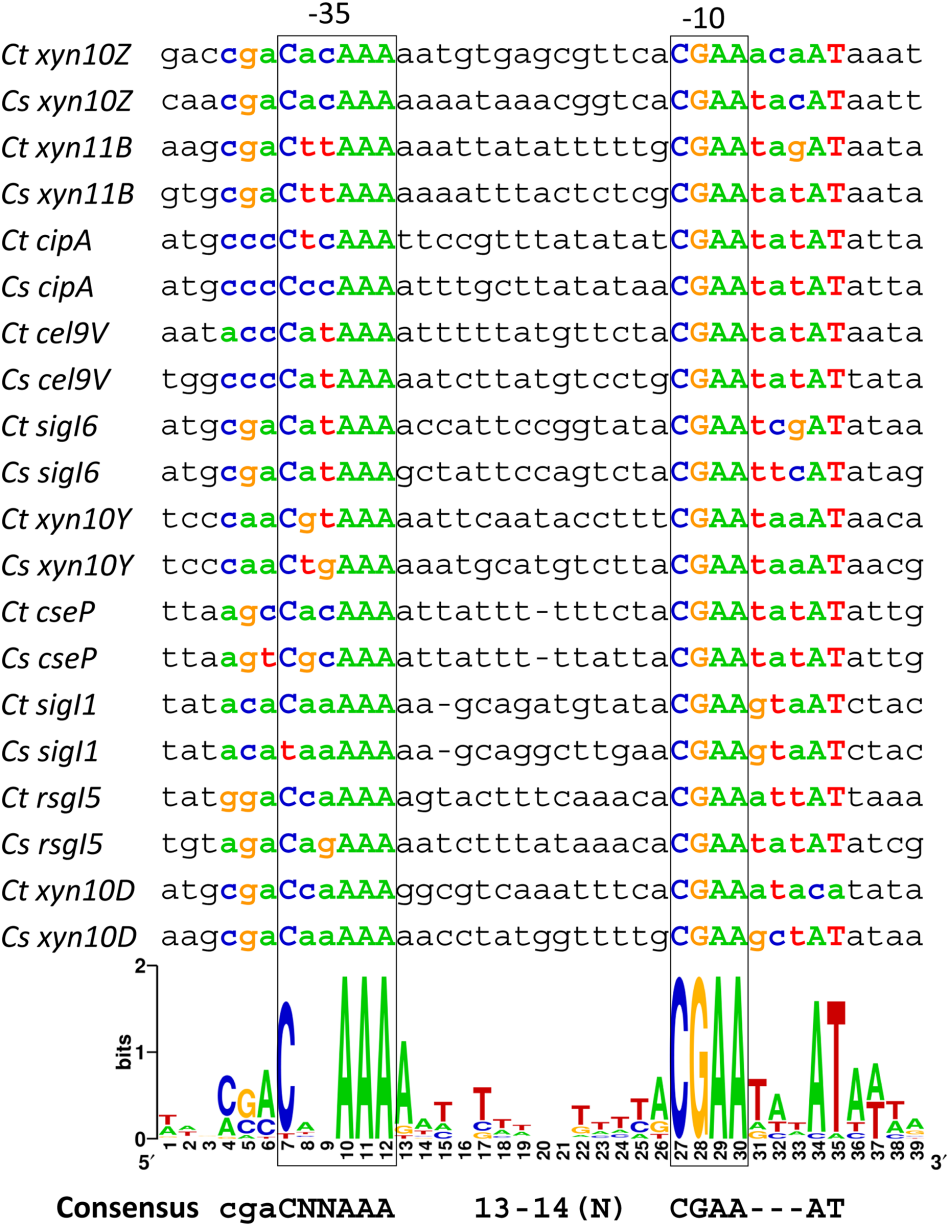
Identification of conserved elements of σ^I6^-dependent promoter sequences. WebLogo generated with σ^I6^-dependent promoter sequences of *C*. *thermocellum* and orthologous promoter sequences of *C*. *straminisolvens*.

In order to identify the essential bases for σ^I6^ recognition, we performed an alignment using the experimentally validated promoter sequences recognized by *C*. *thermocellum* σ^I6^. To improve the analysis, the 10 experimentally validated σ^I6^-dependent promoter sequences of *C*. *thermocellum* DSM 1313 were compared with orthologous promoter sequences of *C*. *straminisolvens* JCM 21531. The result is shown in Fig 2. It can be observed that the σ^I6^-dependent promoters share two highly conserved sequences. The suggested *C*. *thermocellum* σ^I6^-promoter consensus motifs are CNNAAA for the -35 element and CGAA for the -10 element (where N represents any base). The spacing between the suggested -35 and -10 elements is between 13 and 14 nucleotides (Fig 2). It is interesting to note that downstream of the -10 element there is a highly conserved AT sequence.

**Fig 3:**
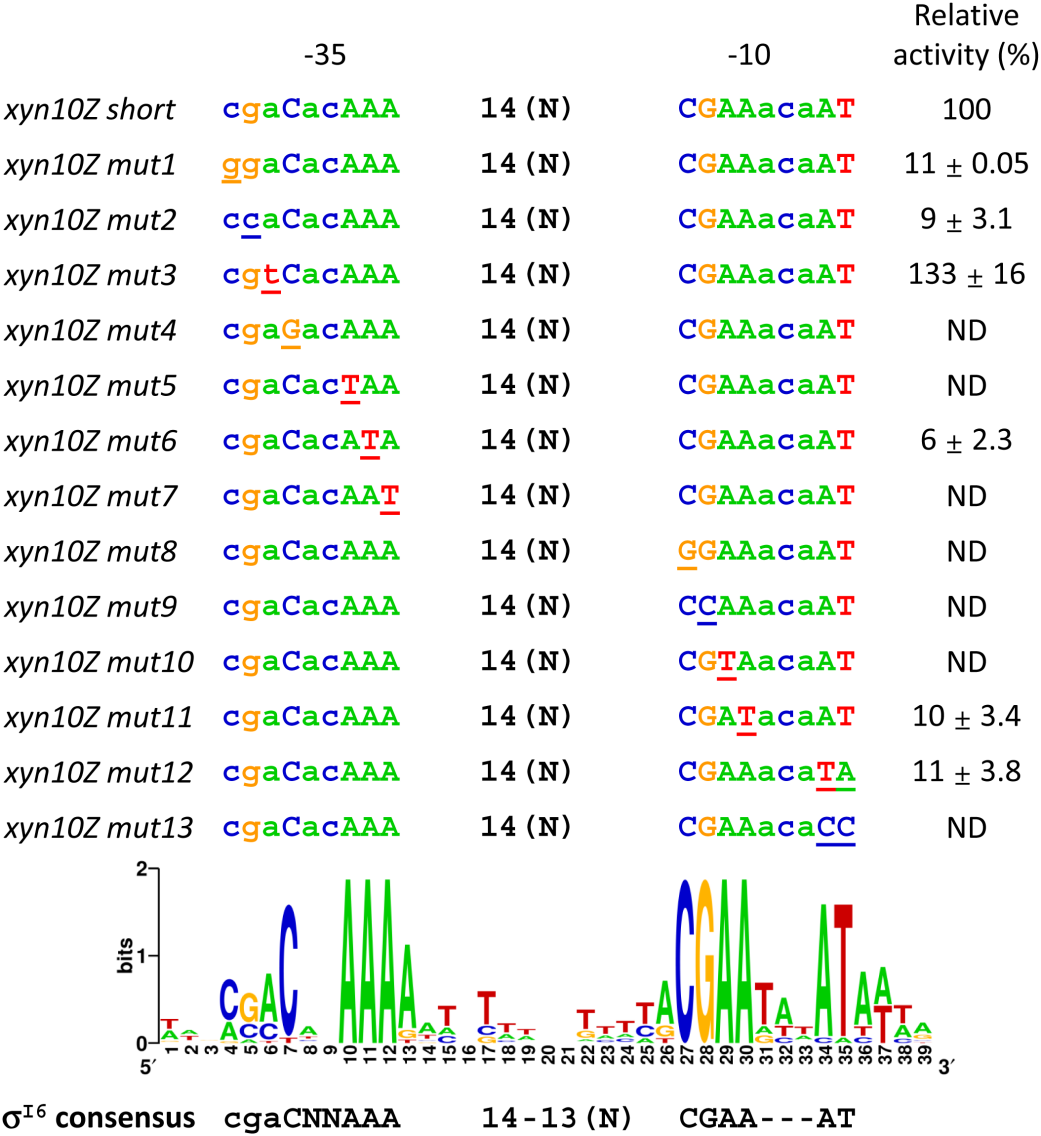
Evaluation of σ^I6^ promoter sequence validity by mutagenesis. The activities are shown as relative activities, with the control promoter *xyn10Zshort* without mutations set to 100%. ND means not detected.

In the -35 element, next to the highly conserved C, a less conserved CGA sequence can be observed in the WebLogo generated with the 10 σ^I6^-dependent promoter sequences of *C*. *thermocellum* and *C*. *straminisolvens* (Fig 2). Interestingly, this CGA sequence is present in the promoter sequences of *sigI6*, *xyn10D*, and in the sequences of the two strongest promoters identified, *xyn10Z* and *xyn11B* (Fig 2 and Table 3). In order to analyze the importance of these bases for the recognition by *C*. *thermocellum* σ^I6^, we performed site-directed mutagenesis analysis using the promoter sequence of *xyn10Z*. Additionally, we evaluated the most conserved bases, which are suggested as the *C*. *thermocellum* σ^I6^-promoter consensus, including the highly conserved AT sequence downstream of the -10 element. The analysis is shown in Fig 3. It can be seen that individual changes in the nucleotides C and G of the CGA sequence (*xyn10Z mut1* and *mut2*) at the 5' of the -35 element reduced dramatically the LacZ activity. Interestingly, the mutation from A to T in the CGA sequence (*xyn10Z mut3*) increased the LacZ activity by 33%. This result shows that although the nucleotides C and G in the CGA sequence of the -35 element is less conserved, they play an important role in recognition by *C*. *thermocellum* σ^I6^.

As expected, individual mutations in the highly conserved nucleotide C (*xyn10Z mut4*) and in the AAA sequence (*xyn10Z mut5*, *mut6* and *mut7*) of the -35 element abolished or reduced dramatically the LacZ activity. The least "sensitive" nucleotide was the middle A (*xyn10Z mut6*) in the AAA triplet of the -35 element. Regarding the -10 element, the most conserved bases were also highly sensitive to mutations. Individual changes in the highly conserved CGAA sequence showed that the first 3 nucleotides CGA (*xyn10Z mut10*, *mut11* and *mut13*) are more sensitive to changes than the last A at the 3' of the CGAA sequence (*xyn10Z mut14*). Finally, changes of the highly conserved AT sequence at the 3' of the -10 element showed a dramatic reduction of LacZ activity when the sequence was changed to TA (*xyn10Z mut12*). However, when the AT sequence was changed to CC, LacZ activity was not detected (*xyn10Z mut13*). All these results confirm the importance of the highly conserved sequences of the -35 and -10 elements, as well as the less conserved nucleotides C and G of the CGA sequence at the 5' of the -35 element.

### Searching for σ^I3^–dependent promoter sequences of cellulosomal genes in *C*. *thermocellum*

In this work, we developed a new methodology that employs *B*. *subtilis* as a heterologous host to verify *C*. *thermocellum* σ^I^-dependent promoters. By exploiting this approach, we extended the promoter analysis to other *C*. *thermocellum* σ^I^ factors. The above-mentioned 40 predicted σ^I^-dependent promoters (Tables 1 and 2) were also analyzed with *C*. *thermocellum* σ^I3^ expressed in *B*. *subtilis*. Table 4 shows LacZ activity of the four predicted promoters that were recognized by *C*. *thermocellum* σ^I3^. These promoters were deduced upstream of the *C*. *thermocellum* genes *sigI3*, *pl11*, *ce12* and *cipA*. As expected, *C*. *thermocellum* σ^I3^ was able to recognize its own promoter, again suggesting autoregulation in *C*. *thermocellum*. Two of the predicted promoters recognized by *C*. *thermocellum* σ^I3^ belong to genes coding for pectin-degrading enzyme (*pl11* and c*e12*). Furthermore, during the quantification of σ^I3^-dependent promoter activities, the promoters of *pl11* and *ce12* showed the highest activities (Table 4). Interestingly, the previously verified σ^I6^-dependent promoter of *cipA* was also recognized by *C*. *thermocellum* σ^I3^ (but to a lesser degree) suggesting a possible overlap between the regulons of *C*. *thermocellum* σ^I3^ and σ^I6^ (Tables 3 and 4).

**Table 4:** Quantitative evaluation of possible promoters under the control of *C*. *thermocellum* σ^I3^.

Gene	Locus tags^a^	Gene product^b^	Ref.	Activity (U)^d^
*pl11*	Clo1313_1983, Cthe_0246	Cellulosomal pectinase:Doc-CBM35-RGL11	----	195 ± 13
*ce12*	Clo1313_0693, Cthe_3141	Cellulosomal pectinase:RGAE-Doc-CBM35-RGAE	----	62 ± 8
*cipA*	Clo1313_0627, Cthe_3077	Cellulosomal scaffoldin subunit:2(Coh)-CBM3-6(Coh)-X-Doc	[45]	58 ± 7
*sigI3*	Clo1313_1911, Cthe_0315	Alternative σ^I3^ factor	[17]	28 ± 13
*sigI6*^c^	Clo1313_2778, Cthe_2120	Alternative σ^I6^ factor	[17]	ND

^*a*^ Clo1313 and Cthe are the locus tag prefixes of *C*. *thermocellum* strains DSM 1313 and ATCC 27405, respectively.

^*b*^ CE, carbohydrate esterase; CBM, carbohydrate binding module; Doc, dockerin; RGL, rhamnogalacturonan lyase; RGAE, rhamnogalacturonan acetylesterase Coh, cohesin; X, CipA X-module; UNK, unknown domain.

^*c*^ The predicted promoter of *C*. *thermocellum sigI6* was used as negative control.

^*d*^ The activity was measured using MUG as substrate. One unit of enzyme activity (U) was defined as the amount of β-galactosidase that releases 1 μmol of 4-MU per minute Numbers in parenthesis represent the standard deviation of at least three independent experiments. ND means not detected.

**Fig 4:**
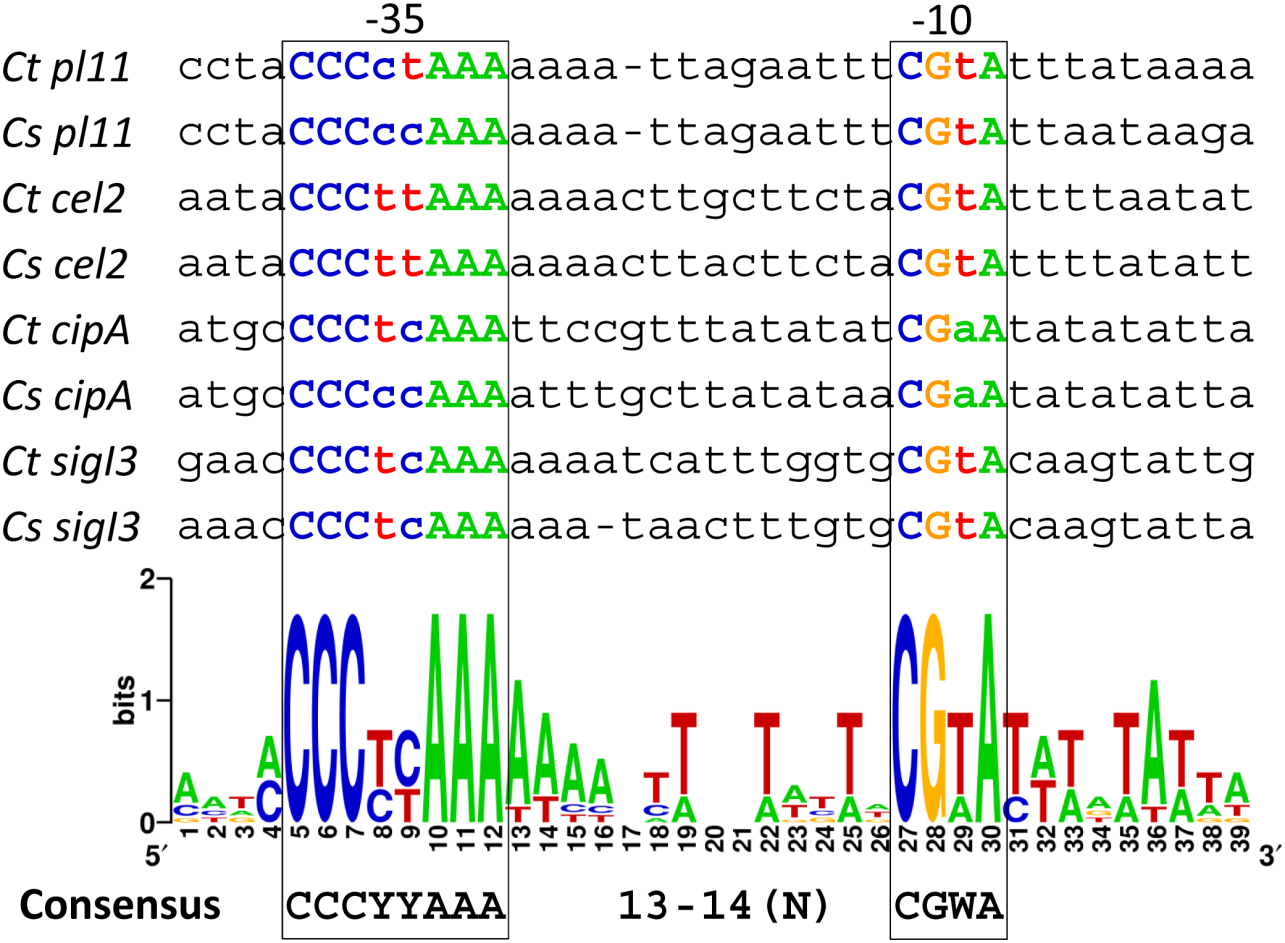
Identification of conserved elements of σ^I3^-dependent promoter sequences. WebLogo generated with σ^I3^-dependent promoter sequences of *C*. *thermocellum* and orthologous promoter sequences of *C*. *straminisolvens*.

In order to identify the important promoter nucleotides for σ^I3^ recognition, we performed an alignment using the experimentally validated promoter sequences recognized by *C*. *thermocellum* σ^I3^. To improve the analysis, the four experimentally validated σ^I3^-dependent promoter sequences were compared with orthologous promoter sequences of *C*. *straminisolvens*. The result is shown in Fig 4. It can be observed that σ^I3^-dependent promoters have two highly conserved sequences. The suggested *C*. *thermocellum* σ^I3^-promoter consensus is CCCYYAAA for the -35 element and CGWA for the -10 element (where Y represents C or T, and W represents A or T). The spacing between the -35 and -10 elements is between 13 and 14 nucleotides, resembling the organization of the σ^I6^ promoter (Fig 2).

## Discussion

Since the original discovery of the cellulosome, numerous observations indicated that its production and composition is influenced by the nature of the carbon source present in the growth media [7,50]. However, until now, there are only a few reports in the literature regarding the regulation of cellulosomal genes [51–54]. Most of these studies were focused on key cellulosomal genes, such as *cipA* [51], *cel48S* [52], *cel8A* [53], *cel9D* [54] and *cel9F* [54]. In the majority of these works, the authors were able to identify σ^A^-dependent promoters upstream of the analyzed genes [51–54]. Additionally, these genes appeared to be regulated by alternative σ factors [51–54]. Nonetheless, the assignment of alternative σ factors was problematic, and in the case of *cel9D* [54] and *cel9F* [54], the authors could not suggest a convincing alternative σ factor. This obstacle surfaced since basic knowledge about *C*. *thermocellum* was limited, and the DNA sequence upstream of the start sites did not contain homologies with described consensus promoters [54].

In an effort to gather knowledge about the regulation of cellulosomal genes, our research group discovered a set of eight alternative σ^I^ factors [17] where six of them showed up-regulation by environmental polysaccharides [19]. This set of *C*. *thermocellum* σ^I^ factors is homologous to the *B*. *subtilis* σ^I^ factor [17,18]. Hence, in order to identify the important *C*. *thermocellum* σ^I^ promoter elements, in the present work, we decided to compare the upstream regions of *C*. *thermocellum* σ^I^ factor genes with the *Bacillales* σ^I^-dependent promoter sequences. The high conservation of the *Bacillales* σ^I^-dependent promoter sequences identified in this study indicated that the *C*. *thermocellum sigI-rsgI* operons may likely have similar -35 and -10 promoter elements. Additionally, it was interesting that the *Bacillales* σ^I^-dependent promoters have a C "enrichment" in their -35 elements (Fig 1A). This observation suggested an easy way to search for σ^I^-dependent promoter candidates in other species of the *Firmicutes* phylum, and notably, for our purposes, the cellulosome-producing bacteria, especially since one striking characteristic of the *Firmicutes* phylum is the low G/C content of their genomes [55]. However, during the search of putative σ^I^-dependent promoters in *C*. *thermocellum*, only *sigI2* and *sigI3* showed the anticipated high C-enrichment in the -35 element motif (Table 1). Subsequently, we compared the *C*. *thermocellum sigI2* and *sigI3* predicted promoter sequences with those of *C*. *thermocellum sigI1* and *sigI6* genes proposed by Nataf and co-workers [19]. This comparison revealed that the promoter regions of *sigI1*, *sigI2*, *sigI3* and *sigI6* have specific "signatures", such as an AAA triad at the -35 region and a CGWA tetrad at the -10 region (Table 1).

Using these specific "signatures", we were able to predict the putative promoters of *C*. *thermocellum sigI4*, *sigI7* and *sigI8*. Moreover, the conservation of these specific "signatures" within σ^I^-dependent promoters was additionally supported by the orthologous *sigI*-promoter sequences of the closely related cellulosome-producing bacterium, *C*. *straminisolvens* JCM 21531 (Table 1). Furthermore, these specific "signatures" were corroborated with the predicted promoter sequences of *sigI*-like genes of *C*. *clariflavum*, *A*. *cellulolyticus* and *P*. *cellulosolvens* and the currently identified 33 σ^I^-dependent promoters of *C*. *thermocellum* cellulosomal genes (Table 2 and S5 Table).

The predicted promoter sequences of the σ^I^-dependent promoters of cellulosome-producing bacteria can be divided into three regions. Two regions are highly conserved and contain the proposed specific "signatures" of the σ^I^-dependent promoters, namely the AAA triad and the CGWA tetrad sequences of the "extended -35" and -10 element, respectively (Tables 1 and 2, and Fig 1). The third region is highly divergent and is located in the 5'-terminal sequence of the -35 elements (corresponding to the *B*. *subtilis* ACCCC sequence, Tables 1 and 2 and Fig 1). We predict that, whereas the most conserved sequences of the "extended -35" and -10 elements are implicated in the "general" recognition of promoters by their cognate σ^I^ factors, the most divergent 5'-terminal sequence of the -35 elements is likely implicated in the specificity of the different σ^I^ factors. This phenomenon could reflect the general strategy followed by σ^I^ factors in order to recognize their target promoters in cellulosome-producing bacteria, whose genomes encode multiple σ^I^ factors. Our hypothesis was herein supported by experimental identification of the putative *C*. *thermocellum* σ^I6^- and σ^I3^-dependent promoters (Figs 2 and 3). Whereas the *C*. *thermocellum* σ^I6^-dependent promoters have a highly conserved C nucleotide upstream of the AAA in the -35 element, the *C*. *thermocellum* σ^I3^-dependent promoters have a highly conserved CCC triad (Figs 2 and 3). Additionally, the analysis of the *C*. *thermocellum* σ^I6^- and σ^I3^-dependent promoters indicated that, in addition, some nucleotides in the -10 element probably have an important role in the specificity of the different σ^I^ factors of cellulosome-producing bacteria. For example, all of the identified *C*. *thermocellum* σ^I6^-dependent promoters have the 5'-located CGAA sequence in their -10 elements, and most of the promoters have an AT sequence three bases downstream (Fig 2). Regarding the identified *C*. *thermocellum* σ^I3^-dependent promoters, they have a less conserved -10 element with the sequence CGWA, where W could be A or T (Fig 3).

Four of the 10 promoters that were activated by *C*. *thermocellum* σ^I6^ belong to genes implicated in the hydrolysis of xylan (*xyn10Z*, *xyn11B-xyn11A* operon, *xyn10Y* and *xyn10D*). This observation is in accordance with previous experiments performed by Nataf and co-workers [19] which showed that when *C*. *thermocellum* was grown on cellulose, the expression of the *sigI6* gene was up-regulated 2.5-fold; and when the cells were grown on cellulose and xylan, *sigI6* was up-regulated at least 10-fold. Moreover, the anti-σ^I6^ factor, RsgI6, bears an extracytoplasmic C-terminal sensing module that belongs to the glycoside hydrolase family 10 (GH10). Interestingly, the RsgI6 GH10-family module is highly similar to Xyn10D (it is 57% identical and has 79% similar residues in the 381-aa gapless alignment; data not shown). Bahari and co-workers [21] showed that the RsgI6 GH10-like domain binds to oat-spelt xylan and Avicel (cellulose). Furthermore, a recent study performed by Wei and co-workers [16], showed that the genes *xyn10Y* and *xyn10D* were up-regulated when *C*. *thermocellum* was grown in dilute acid-pretreated yellow poplar, containing 65% cellulose, 4% xylan and 31% lignin.

Regarding *C*. *thermocellum* σ^I3^, two of the four promoters that were activated by *C*. *thermocellum* σ^I3^ belong to genes implicated in the solubilization of pectin (*pl11*/Clo1313_1983 and *ce12*/Clo1313_0693). Interestingly, the anti-σ^I3^ factor RsgI3, has an extracytoplasmic sensing module that is composed of two tandem PA14 superfamily motifs that were shown to bind pectin by Kahel-Raifer and co-workers [17]. Taken together, these results suggest that while σ^I6^ likely plays a role in the regulation of xylan-degrading enzymes, σ^I3^ likely plays a role in the regulation of pectin-degrading enzymes.

The *C*. *thermocellum sigI1* and *rsgI5* genes are part of the proposed genes involved in the regulation of cellulosomal genes in response to environmental polysaccharides [17]. In the present work, the two predicted σ^I^-dependent promoters for *sigI1* and *rsgI5* were recognized by *C*. *thermocellum* σ^I6^ (Table 3). Furthermore, the predicted σ^I^-dependent promoter for *cipA* was recognized by both *C*. *thermocellum* σ^I6^ and σ^I3^ (Tables 3 and 4). These results suggest possible crosstalk between different *C*. *thermocellum* σ^I^ factors and a possible overlap of their respective regulons. Interestingly, this phenomenon is common in ECF sigma factors. For example, Huang and co-workers [56] found in *B*. *subtilis* that σ^W^ recognizes a subset of promoters that are partially dependent on σ^X^ for expression. Additionally, Mascher and co-workers [57] found 7 ECF sigma factors in *B*. *subtilis* that regulate partially overlapping regulons related to cell envelope homeostasis and antibiotic resistance. To unwrap its preferred substrate, cellulose, that is covered with different types of polysaccharides in the plant cell wall, *C*. *thermocellum* would presumably produce an array of different hydrolytic cellulosomal components. Hence, to have partially overlapping regulons for the multiple σ^I^ factors could be advantageous, because expression of a variety of cellulosomal components is crucial for efficient solubilization of the different types of polysaccharides that conceal the cellulose fibers in their native state.

Our results and observations reveal several promising options to improve the performance of the industrially prominent bacterium *C*. *thermocellum*. First of all, by changing promoter designs by metabolic engineering, we may try to modify the expression of selected cellulosomal genes that might be crucial for production of natural forms of designer cellulosomes. Secondly, by using strong sigma-dependent promoters (e.g., those of *xyn10Z*, *xyn11B*, *pl11*, etc.) one can introduce additional, synthetic cellulosomal genes in *C*. *thermocellum* and use their products for improvement of either saccharolytic activity or, alternatively, ethanol production. Continued analysis and harnessing of the various σ and anti-σ factors in *C*. *thermocellum* will allow us to control and enhance the capacity of this ecologically prominent and industrially relevant bacterium for deconstruction of plant-derived polysaccharides *en route* to the production of biofuels.

## Supporting Information

S1 Fig Schematic depiction of plasmid pLOXErysigIrsgIBs. (PDF)

S2 Fig ClustalW alignment of the RNAP subunits sequences of *Bacillus subtilis* strain 168 and *Clostridium thermocellum* strain DSM 1313. (PDF)

S1 Table Primers used in the present work. (PDF)

S2 Table Plasmids constructed in the present work. (PDF)

S3 Table *Bacillus subtilis* strains constructed in the present work. (PDF)

S4 Table Alignment of experimentally confirmed and putative *sigI* promoters from different *Bacillales* species, including the experimentally confirmed *sigI*-dependent promoters of the *B*. *subtilis bcrC* and *mreBH* genes. (PDF)

S5 Table Alignments of predicted *sigI* promoters from *Clostridium clariflavum*, *Acetivibrio cellulolyticus*, *Pseudobacteroides cellulosolvens*, *Clostridium thermocellum* and *Clostridium straminisolvens*. (PDF)

S6 Table σ^I6^-dependent promoter sequence alignment of *xyn11B-xyn11A* operon and *xyn11A* of available sequences of *C*. *thermocellum* and C. *straminisolvens* JCM 21531. (PDF)
