# Supplementary figures and images for: Decoding Biomass-Sensing Regulons of Clostridium thermocellum Alternative Sigma-I Factors in a Heterologous Bacillus subtilis Host System
Source: PLoS One. 2016 Jan 5;11(1):e0146316. doi: 10.1371/journal.pone.0146316 (PMC4711584; doi:10.1371/journal.pone.0146316)

S1 Figure. Schematic depiction of plasmid pLOXErysigIrsglBs.

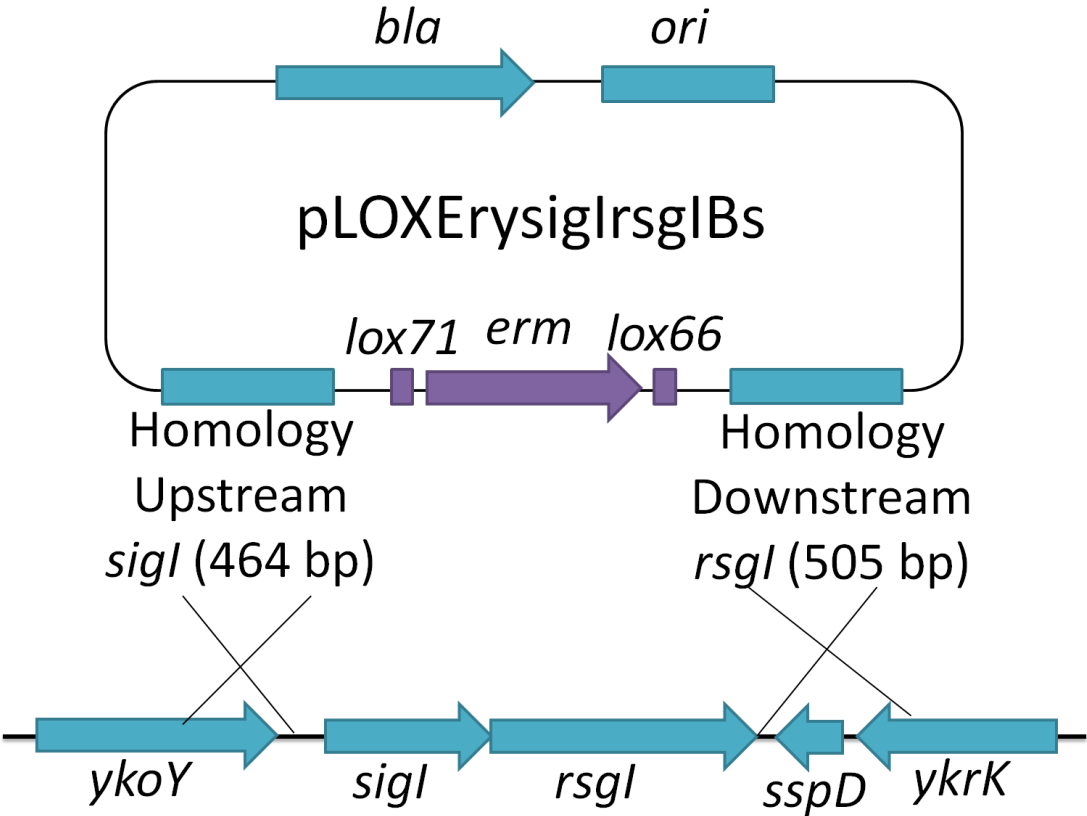

Supplement: S1 Fig — (PDF) [file pone.0146316.s001.pdf]
