# Supplementary material for: Decoding Biomass-Sensing Regulons of Clostridium thermocellum Alternative Sigma-I Factors in a Heterologous Bacillus subtilis Host System
Source: PLoS One. 2016 Jan 5;11(1):e0146316. doi: 10.1371/journal.pone.0146316 (PMC4711584; doi:10.1371/journal.pone.0146316)
Supplement: S2 Fig — (PDF) [file pone.0146316.s002.pdf]

S2 Figure. **ClustalW alignment of the RNAP subunits sequences of *Bacillus subtilis* strain 168 and *Clostridium thermocellum* strain DSM 1313**.

Alignments were performed by using an on-line server:
https://npsa-prabi.ibcp.fr/cgi-bin/npsa_automat.pl?page=/NPSA/npsa_clustalw.html

σ^A (RpoD) subunit

```
                         10        20        30        40        50        60
B_subtilis       MADKQTHETELTFDQVKEQLTESGKKRGVLTYEEIAERMSSFEIESDQMDEYYEFLGEQG
C_thermocellum   MKPNS--DKK----AILLELIEKGKQKGMLTYQEIMDAFEEVDIDPEQIEKIYETLENMG
                 *  :.  :.:     :  :* *.**::*:***:** : :...:*:.:*::: ** * : *

                         70        80        90       100       110       120
B_subtilis       VELISENEETEDPNIQQLAKAEEEFDLNDLSVPPGVKINDPVRMYLKEIGRVNLLSAKEE
C_thermocellum   IDVVGDIE-AE---MEDIQLTEDDLDL---SIPEGISIDDPVRMYLKEIGKVPLLTAEEE
                 ::::.: * :*   ::::  :*:::**   *:* *:.*:***********:* **:*:**

                        130       140       150       160       170       180
B_subtilis       IAYAQKIEEGDEESKRRLAEANLRLVVSIAKRYVGRGMLFLDLIQEGNMGLMKAVEKFDY
C_thermocellum   IELAHRMEQGDAEAKRRLAEANLRLVVSIAKRYVGRGMLFLDLIQEGNLGLIKAVEKFDY
                 *  *:::*:** *:**********************************:**:********

                        190       200       210       220       230       240
B_subtilis       RKGYKFSTYATWWIRQAITRAIADQARTIRIPVHMVETINKLIRVQRQLLQDLGREPTPE
C_thermocellum   RKGFKFSTYATWWIRQAITRAIADQARTIRIPVHMVETINKLIRVSRQLLQELGREPHPE
                 ***:*****************************************.*****:***** **

                        250       260       270       280       290       300
B_subtilis       EIAEDMDLTPEKVREILKIAQEPVSLETPIGEEDDSHLGDFIEDQEATSPSDHAAYELLK
C_thermocellum   EIAKEMNMPVEKVREIMKISQEPVSLETPIGEEEDSHLGDFIPDDDAPAPSEAAAFTLLK
                 ***::*::. ******:**:*************:******** *::*.:**: **: ***

                        310       320       330       340       350       360
B_subtilis       EQLEDVLDTLTDREENVLRLRFGLDDGRTRTLEEVGKVFGVTRERIRQIEAKALRKLRHP
C_thermocellum   EQLVDVLDTLTPREEKVLRLRFGLDDGRARTLEEVGKEFNVTRERIRQIEAKALRKLRHP
                 *** ******* ***:************:******** *.********************

                        370
                          |
B_subtilis       SRSKRLKDFLE
C_thermocellum   SRSKKLKDYLD
                 ****:***:*:
```

Alignment length: 371; **identical** (*): 253 is **68.20%**; **strongly similar** (:): 64 is 17.25%; **weakly similar** (.): 12 is 3.23%; **different**: 42 is **11.32%**.

α (RpoA) subunit

```
                         10        20        30        40        50        60
B_subtilis       MIEIEKPKIETVEISDDAKFGKFVVEPLERGYGTTLGNSLRRILLSSLPGAAVTSIQIDG
C_thermocellum   MIEIEKPKIECVVCSEDNRYGKFVVEPLERGYGITLGNSLRRILLSSLPGVAVTSIKIDG
                 ********** *  *:* ::************* ****************.*****:***

                         70        80        90       100       110       120
B_subtilis       VLHEFSTIEGVVEDVTTIILHIKKLALKIYSDEEKTLEIDVQGEGTVTAADITHDSDVEI
C_thermocellum   VLHEFSTIPGVIEDVTEIILNIKELSLNFHGEGPKVIYIDAEGEGEVKAKDIKADADVEI
                 ******** **:**** ***:**:*:*:::.:  *.: **.:*** *.* **. *:****
```

```
                         130       140       150       160       170       180
B_subtilis        LNPDLHIATLGENASFRVRLTAQRGRGYTPADANKRDDQPIGVIPIDSIYTPVSRVSYQV
C_thermocellum    LNPEHKIATLSGDHRLYMEMTIDKGRGYVSAEKNKHPGQPIGVIPVDSIFTPVHKVNYTV
                  ***: :****. :  : :.:* ::****..*: **: .*******:***:*** :*.* *

                         190       200       210       220       230       240
B_subtilis        ENTRVGQVANYDKLTLDVWTDGSTGPKEAIALGSKILTEHLNIFVGLTDEAQHAEIMVEK
C_thermocellum    ENTRVGQVTDYDKLTLEVWTNGSIKPDEAISLGAKILSEHLNLFIDLSDNAKNAEIMVEK
                  ********::******:***:**  *.***:**:***:****:*:.*:*:*::*******

                         250       260       270       280       290       300
B_subtilis        EEDQKEKVLEMTIEELDLSVRSYNCLKRAGINTVQELANKTEEDMMKVRNLGRKSLEEVK
C_thermocellum    EETKKEKVLEMTIEELDLSVRSYNCLKRAGINTVEDLISRTEEDMMKVRNLGRKSLEEVV
                  ** :******************************::* .:*******************

                         310
B_subtilis        AKLEELGLGLRK-DD
C_thermocellum    NKLKALGLSLAPSED
                   **: ***.*   :*
```

Alignment length: 315; **identical** (*): 219 is **69.52 %**; **strongly similar** (:): 47 is **14.92 %**; **weakly similar** (.): 16 is **5.08 %**; different: 33 is 10.48 %.

β (RpoB) subunit

```
                          10        20        30        40        50        60
B_subtilis        MTGQLVQYGRHRQRRSYARISEVLELPNLIEIQTSSYQWFLDEGLREMFQDISPIEDFTG
C_thermocellum    MV-HPVKLGRN-VRMSYSKIDEVIDMPNLIEIQKNSYEQFLKEGFKEVFKDVNPITDYTG
                  *  : *: **:  * **::*.**:::*******..**: **.**::*:*:*:.** *:**

                          70        80        90       100       110       120
B_subtilis        NLSLEFIDYSLGEP-KYPVEESKERDVTYSAPLRVKVRLINKETGEVKDQDVFMGDFPIM
C_thermocellum    NLILEFVDYSLDEPPKYSVDECKERDATYAAPLKVKVRLINKETGEVKEQEIFMGDFPLM
                  ** ***:****.** **.*:*.****.**:***:**************:*::******:*

                         130       140       150       160       170       180
B_subtilis        TDTGTFIINGAERVIVSQLVRSPSVYFSGKVDKNGKKGFTATVIPNRGAWLEYETDAKDV
C_thermocellum    TETGTFIINGAERVIVSQLVRSPGIYYAMKIDKAGKQLFSNTVIPNRGAWLEYETDSNDV
                  *:*********************.:*:: *:** **: *: ***************::**

                         190       200       210       220       230       240
B_subtilis        VYVRIDRTRKLPVTVLLRALGFGSDQEILDLIGENEYLRNTLDKDNTENSDKALLEIYER
C_thermocellum    LSVRIDRTRKLPLTVLVRALGYGTDLEITELFGEDERILATIQKDSTKTEEEGLLEIYKR
                  : **********:***:****:*:* ** :*:**:* :  *::**.*:..::.*****:*

                         250       260       270       280       290       300
B_subtilis        LRPGEPPTVENAKSLLDSRFFDPKRYDLANVGRYKINKKLHIKNRLFNQRLAETLVDPET
C_thermocellum    LRPGEPPTVESAKALLHGLFFDPKRYDLAKPGRFKFNKKLSIAARIHGFIAGENIKDPDT
                  **********.**:**.. **********: **:*:**** * *:..   .*.: **:*

                         310       320       330       340       350       360
B_subtilis        GEILAEKGQILDRRTLDKVLPYLENGIGFRKLYPNGGVV-EDEVTLQSIKIFAPTDQ---
C_thermocellum    GEIIVAEGETISREKAETIQNAGVNTVILRVDGKNVKVIGNDMVDIKRYVDFDPKEIGIN
                  ***:. :*: :.*.. :.:     * : :*    *  *: :* * ::    * *.:

                         370       380       390       400       410       420
B_subtilis        -----------------EGEQVINVIGNAYIEEEI-KNITPADIISSISYFFNLLHGVGD
C_thermocellum    EKVKRDVLMEILEEYKGKGDDAIKKALQERIDDLIPKHITKEDIISSISYIIGLSYGIGS
                                   :*::.*:   :  *:: * *:**  ********::.* :*:*.
```

```
                         430       440       450       460       470       480
B_subtilis        TDDIDHLGNRRLRSVGELLQNQFRIGLSRMERVVRERMSIQDTNTITPQQLINIRPVIAS
C_thermocellum    TDDIDHLGNRRLRSVGELLQNQFRIGLSRMERVVRERMTIQDLDVVTPQALINIRPVAAA
                  **************************************:*** :.:*** ******* *:

                         490       500       510       520       530       540
                           |         |         |         |         |         |
B_subtilis        IKEFFGSSQLSQFMDQTNPLAELTHKRRLSALGPGGLTRERAGMEVRDVHYSHYGRMCPI
C_thermocellum    IKEFFGSSQLSQFMDQTNPLAELTHKRRLSALGPGGLSRERAGFEVRDVHHSHYGRMCPI
                  *************************************:*****:******:*********

                         550       560       570       580       590       600
B_subtilis        ETPEGPNIGLINSLSSYAKVNRFGFIETPYRRVDP-ETGKVTGRIDYLTADEEDNYVVAQ
C_thermocellum    ETPEGPNIGLIGSLSTYARVNEYGFIETPYRKVSKEEPGKVTNEIVYLTADEEDEYIIAQ
                  ***********.***:**:**.:********:*.  *.****..* ********:*::**

                         610       620       630       640       650       660
B_subtilis        ANARLDDEGAFIDDSIVARFRGENTVVSRNRVDYMDVSPKQVVSAATACIPFLENDDSNR
C_thermocellum    ANEPLDEEGRFISNKVVCRYKEEFIEVDPSKIDFMDVSPKQIVSVATSMIPFLENDDANR
                  **  **:** **.:.:*.*:: *   *. .::*:*******:**.**: ********:**

                         670       680       690       700       710       720
B_subtilis        ALMGANMQRQAVPLMQPEAPFVGTGMEYVSGKDSGAAVICKHPGIVERVEAKNVWVRRYE
C_thermocellum    ALMGANMQRQAVPLIKTESPIVGTGIEYRAARDSGVVILAKNPGVVEKVTANEIIIR---
                  **************::.*:*:****:** :.:***..::.*:**:**:* *::: :*

                         730       740       750       760       770       780
B_subtilis        EVDGQKVKGNLDKYSLLKFVRSNQGTCYNQRPIVSVGDEVVKGEILADGPSMELGELALG
C_thermocellum    -----TKDGKRDTYKLLKYMRSNQGTCINQRPIVKKGEEVEAGDVIADGPSTDNGEIALG
                       . .*: *.*.***::******* ******. *:**  *:::***** : **:***

                         790       800       810       820       830       840
B_subtilis        RNVMVGFMTWDGYNYEDAIIMSERLVKDDVYTSIHIEEYESEARDTKLGPEEITRDIPNV
C_thermocellum    KNVLVGFMTWEGYNYEDAILISERLVKDDVFTSIHIEEYEAEARDTKLGPEDITREIPNV
                  :**:******:********::*********:*********:**********:***:****

                         850       860       870       880       890       900
B_subtilis        GEDALRNLDDRGIIRIGAEVKDGDLLVGKVTPKGVTELTAEERLLHAIFGEKAREVRDTS
C_thermocellum    SEDALKDLNSEGIIRIGAEVRAGDILVGKVTPKGETELTAEERLLRAIFGEKAREVRDTS
                  .****::*:..*********: **:********* **********:**************

                         910       920       930       940       950       960
B_subtilis        LRVPHGGGGIIHDVKVFNREDGDELPPGVNQLVRVYIVQKRKISEGDKMAGRHGNKGVIS
C_thermocellum    LRVPHGESGIVVDVKIFTRENGDELAPGVNKLVRVYVAQKRKISVGDKMAGRHGNKGVIS
                  ****** .**: ***:*.**:****.****:*****:.****** ***************

                         970       980       990      1000      1010      1020
B_subtilis        KILPEEDMPYLPDGTPIDIMLNPLGVPSRMNIGQVLELHMGMAARYLGIHIASPVFDGAR
C_thermocellum    RILPVEDMPFLPDGTPLDIVLNPLGVPSRMNIGQVLEVHLGYAAKALGWKVATPVFDGAT
                  :*** ****:******:**:*****************:*:* **: ** ::*:******

                        1030      1040      1050      1060      1070      1080
B_subtilis        EEDVWETLEEAGMSRDAKTVLYDGRTGEPFDNRVSVGIMYMIKLAHMVDDKLHARSTGPY
C_thermocellum    EEDIVQTLRKAGLAEDGKSILYDGRTGEPFENRVTVGYMYMLKLAHLVDDKIHARSTGPY
                  ***: :**.:**::.*.*::**********:***:** ***:****:****:********

                        1090      1100      1110      1120      1130      1140
B_subtilis        SLVTQQPLGGKAQFGGQRFGEMEVWALEAYGAAYTLQEILTVKSDDVVGRVKTYEAIVKG
C_thermocellum    SLVTQQPLGGKAQFGGQRFGEMEVWALEAYGAAYTLQEILTVKSDDVVGRVKTYEAIVKG
                  ************************************************************
```

```
                            1150      1160      1170      1180      1190      1200
B_subtilis        DNVPEPGVPESFKVLIKELQSLGMDVKILSGDEEEIEMRD------------LEDEED--
C_thermocellum    ENVPEPGIPESFKVLIKELQSLCLDVKVYSEEQEEIAIKESVEDDLEELNVNIEGREDEV
                  :******:************** :***: * ::*** :::            :*..**

                            1210      1220      1230      1240      1250      1260
B_subtilis        ------------------AKQADGLALSGDEE-PEETASADVERDVVTKE----------
C_thermocellum    NFNEFNDIGEEITDEDLEVEDFDLQDLNDDDINPDDTIDAELDDNLFDDDFDDTFDDDDL
                                    .:: *   *..*:  *::* .*::: ::. .:
```

Alignment length: 1260; **identical** (*): 811 is **64.37%**; **strongly similar** (:): 192 is **15.24%**; **weakly similar** (.): 71 is **5.63%**; **different**: 186 is **14.76%.**

β’ (RpoC) subunit

```
                            10        20        30        40        50        60
B_subtilis        MLDVNNFEYMNIGLASPDKIRSWSFGEVKKPETINYRTLKPEKDGLFCERIFGPTKDWEC
C_thermocellum    MFELNNFDSIRIGLASPEKIREWSRGEVKKPETINYRTLKPERDGLFCERIFGPQKDWEC
                  *:::***: :.******:***.** *****************:*********** *****

                            70        80        90       100       110       120
B_subtilis        HCGKYKRVRYKGVVCDRCGVEVTRAKVRRERMGHIELAAPVSHIWYFKGIPSRMGLVLDM
C_thermocellum    HCGKYKRIRYKGIVCDRCGVEVTRSKVRRERMGHIELAAPVSHIWYFKGIPSRMGLLLDM
                  *******:****:***********:*******************************:***

                           130       140       150       160       170       180
B_subtilis        SPRALEEVIYFASYVVTDPANTPLEKKQLLSEKEYRAYLDKYGNKFQASMGAEAIHKLLQ
C_thermocellum    SPRALEKILYFAAYVVIDPGQTPLSKKQILSEKEYRDSLEKFGPKFRAGMGAEAVRELLQ
                  ******:::***:*** **.:***.***:*******  *:*:* **:*.*****:::***

                           190       200       210       220       230       240
B_subtilis        DIDLVKEVDMLKEELKTSQGQRRTRAIKRLEVLEAFRNSGNKPSWMILDVLPVIPPELRP
C_thermocellum    EINLDELSAELREEIKQSTGQKRVRAIKRLEVVEAFRQSQNKPEWMILDVIPVIPPELRP
                  :*:* :     *:**:* * **:*.********:****:* ***.******:*********

                           250       260       270       280       290       300
B_subtilis        MVQLDGGRFATSDLNDLYRRVINRNNRLKRLLDLGAPSIIVQNEKRMLQEAVDALIDNGR
C_thermocellum    MVQLDGGRFATSDLNDLYRRVINRNNRLKRLLDLGAPDIIVRNEKRMLQEAVDALIDNGR
                  *************************************.***:******************

                           310       320       330       340       350       360
B_subtilis        RGRPVTGPGNRPLKSLSHMLKGKQGRFRQNLLGKRVDYSGRSVIVVGPHLKMYQCGLPKE
C_thermocellum    RGRPVTGPGNRPLKSLSDMLKGKQGRFRQNLLGKRVDYSGRSVIVVGPELKIYQCGLPKE
                  *****************.******************************.**:********

                           370       380       390       400       410       420
B_subtilis        MALELFKPFVMKELVEKGLAHNIKSAKRKIERVQPEVWDVLESVIKEHPVLLNRAPTLHR
C_thermocellum    MALELFKPFVMKKLVNDGLAHNIKSAKRMVERVRNEVWDVLEEVIKEHPVLLNRAPTLHR
                  ************:**:.*********** :***: *******.*****************

                           430       440       450       460       470       480
B_subtilis        LGIQAFEPTLVEGRAIRLHPLVCTAYNADFDGDQMAVHVPLSAEAQAEARILMLAAQNIL
C_thermocellum    LGIQAFEPVLVEGRALKLHPLVCTAYNADFDGDQMAIHVPLSAEAQAEARFLMLSANNLL
                  ********.******::*******************:*************:***:*:*:*

                           490       500       510       520       530       540
B_subtilis        NPKDGKPVVTPSQDMVLGNYYLTLERAGAVGEGMVFKNTDEALLAYQNGYVHLHTRVAVA
C_thermocellum    KPQDGKPVAVPTQDMVLGSYYLTILKEGAKGEGRVFTSMDEAVMAYDNGEIELHSKIKVR
                  :*:*****..*:******.****: : ** *** **..  ***::**:** :.**::: *
```

```
                        550       560       570       580       590       600
B_subtilis       ANSLKNVTFTEEQRSKLLITTVGKLVFNEILPESFPYMNEPTKSNIEEKTPDRFFLEKGA
C_thermocellum   MKRVVDG----VEKSKIIETTLGRLIFNEAIPQDLGFVDRSDPDKIFDLEVD--FLVG--
                  : : :      ::**:: **:*:*:*** :*:.: :::..  .:* :   *  **

                        610       620       630       640       650       660
B_subtilis       DVKAVIAQQPINAPFKKGILGKIIAEIFKRFHITETSKMLDRMKNLGFKYSTKAGITVGV
C_thermocellum   ----------------KNELKKIIDKSIKVHGTTKTAILLDKIKELGFKYSTKGAITISI
                                 *. * *** : :* .  *:*: :**::*:********..**:.:

                        670       680       690       700       710       720
B_subtilis       SDIVVLDDKQEILEEAQSKVDNVMKQFRRGLITEEERYERVISIWSAAKDVIQGKLMKSL
C_thermocellum   SDMVIPEVKAKYIKETEEKIEKITKQYKRGLISDEERYNSVIAAWTEASENITRALINNL
                 **:*: : * : ::*::.*:::: **::****::****: **: *: *.: *   *::.*

                        730       740       750       760       770       780
B_subtilis       DELNPIYMMSDSGARGNASNFTQLAGMRGLMANPAGRIIELPIKSSFREGLTVLEYFIST
C_thermocellum   DRFNPVYMMSQSGARGNINQIKQLAGMRGLMADTSGKTIEFPIKANFREGLTVMEFFIST
                 *.:**:****:****** .::.**********:.:*: **:***:.*******:*:****

                        790       800       810       820       830       840
B_subtilis       HGARKGLADTALKTADSGYLTRRLVDVAQDVIIRETDCGTDRGILAKPLKEGTETIERLE
C_thermocellum   HGARKGLADTALRTADSGYLTRRLVDVSQDVIVRETDCGTRKGIEVTDIKDGNEVIEELS
                 ************:**************:****:******* :** .. :*:*.*.**.*.

                        850       860       870       880       890       900
B_subtilis       ERLIGRFARKQVKHPETGEVLVNENELIDEDKALEIVEAGIEEVWIRSAFTCNTPHGVCK
C_thermocellum   ERIIGRYPVGNIVHPETGEIIVEAGRMITDQDAEKIVKAGIKKVRIRSVLTCHSEYGVCA
                 **:***:.  :: ******::*: ..:* ::.* :**:***::* ***.:**:: :***

                        910       920       930       940       950       960
B_subtilis       RCYGRNLATGSDVEVGEAVGIIAAQSIGEPGTQLTMRTFHTGGVAGDDITQGLPRIQELF
C_thermocellum   KCYGANLATGEECNVGEAVGIIAAQSIGEPGTQLTMRTFHTGGVAGEDITQGLPRVEELF
                 :*** *****.: :********************************:********::***

                        970       980       990      1000      1010      1020
B_subtilis       EARNPKGQATITEIDGTVVEINEVRDKQQEIVVQGAVETRSYTAPYNSRLKVAEGDKITR
C_thermocellum   EARKPKGLAIISEIKG-TVKISETKKKREIVVTSEDGETRSYLIPYGSRIKVSDGDQVEA
                 ***:*** * *:**.* .*:*.*.:.*:: :*..   *****  **.**:**::**::

                       1030      1040      1050      1060      1070      1080
B_subtilis       GQVLTEGSIDPKELLKVTDLTTVQEYLLHEVQKVYRMQGVEIGDKHVEVMVRQMLRKVRV
C_thermocellum   GDELTEGSVNPHDILKIKGVEAVQTYLVHEVQKVYRMQGVDINDKHIEVIVRQMLRKVKV
                 *: *****::*:::**:..: :** **:************:*.***:**:********:*

                       1090      1100      1110      1120      1130      1140
B_subtilis       IDAGDTDVLPGTLLDIHQFTEANKKVLLEGNRPATGRPVLLGITKASLETDSFLSAASFQ
C_thermocellum   EDPGDTSLLPGGLVDVFDFEEENAKAIAEGKKPAVAKRALLGITKAALATDSFLSAASFQ
                  *.***.:*** *:*:.:* * * *.: **::**..: .*******:* ***********

                       1150      1160      1170      1180      1190
B_subtilis       ETTRVLTDAAIKGKRDELLGLKENVIIGKLVPAGTGMMKYRKVKPVSNVQPTDDMVPVE
C_thermocellum   ETTRVLTEAAIKGKVDPLVGLKENVIIGKLIPAGTGMSRYKDIT-ISTVTE--------
                 *******:****** * *:***********:****** :*:.:. :*.*
```

Alignment length: 1199; **identical** (*): 802 is **66.89%**; **strongly similar** (:): 191 is **15.93%**; **weakly similar** (.): 69 is **5.75%**; **different**: 137 is **11.43%.**

ω (RpoZ) subunit

```
                         10        20        30        40        50        60
B_subtilis       ----------MLDPSIDSLMNKLDSKYTLVTVSARRAREMQIKKDQMIEHTISHKYVGKA
C_thermocellum   MKEKKERVSSMIEPSINSLLEKVDSRYTLVVATAKRARQLTDGANKLTN-CESDKPVTVA
                           *::***:**::*:**:****..:*:***::    ::: :   *.* *   *

                         70
B_subtilis       LEEIDAGLLSFEKEDRE--
C_thermocellum   INEINENKITYIRTKSGIK
                 ::**: . ::: : .
```

Alignment length: 79; **identical** (*): 23 is **29.11%**; **strongly similar** (:): 22 is **27.85%**; **weakly similar** (.): 5 is **6.33%**; **different**: 29 is **36.71%**.
