# Supplementary material for: Decoding Biomass-Sensing Regulons of Clostridium thermocellum Alternative Sigma-I Factors in a Heterologous Bacillus subtilis Host System
Source: PLoS One. 2016 Jan 5;11(1):e0146316. doi: 10.1371/journal.pone.0146316 (PMC4711584; doi:10.1371/journal.pone.0146316)
Supplement: S1 Table — (PDF) [file pone.0146316.s003.pdf]

S1 Table. Primers used in the present work.

**Primers used for the construction of pLOXErysigIrsgIBs plasmid**			
**#**	**Name**	**Sequence 5'→3'**	**Brief description**
P1[a]	Fw.ykoY.fus	CGGTACCCGGGGATCGAAGATCGGCGGACTTGAC	Amplification upstream region of *Bs sigI*
P2[a]	Rv.ykoY.fus	TCCTTTCTCGCCTGCCGTGTTTTTTGTAACAAAG	
P3[a]	Fw.sspD.fus	GTGACAGGAGCCTCGAGACCTGAATTTATTTAGTTGTGC	Amplification downstream region of *Bs rsgI*
P4[a]	Rv.ykrK.fus	CGACTCTAGAGGATCTGCAGGAGATCGCCGAACAG	
P5[a]	Fw.loxerm	GCAGGCGAGAAAGGAGAG	Amplification *lox71-erm-lox66* cassette
P6[a]	Rv.loxerm	CGAGGCTCCTGTCACTGCTTC	
**Primers used for the amplification of *C. thermocellum* DSM 1313 *sigI6* and *sigI3* genes during the construction of pAX01 derived plasmids**			
**#**	**Name**	**Sequence 5'→3'**	**Brief description**
P7[a]	Fw.sigI6.fus	GGGGGAAATGGGATCATGGATTGGCATTTTCAAGG	Amplification of *Ct sigI6* gene
P8[a]	Rv.sigI6.fus	CGCGGGAGCTCGATCTCACCGCAAATCCACCTCC	
P9[a]	Fw.sigI3.fus	GGGGGAAATGGGATCATGCATGGGTTGTTTGTAAATA	Amplification of *Ct sigI3* gene
P10[a]	Rv.sigI3.fus	CGCGGGAGCTCGGATCCTTAGTTATCCATGATACTTTC	
**Primers used for the amplification of the promoters of *C. thermocellum* DSM 1313 *sigI* factors during the construction of pBS1ClacZ derived plasmids**			
**#**	**Name**	**Sequence 5'→3'**	**Brief description**
P11	Fw.PsigI1.Eco	GAGAATTCAGCAAGAGCCGATATTAATCG	Amplification promoter of *Ct sigI1*
P12	Rv.PsigI1.Bam	TTGGATCCCCAGATCATTCTCCCTTCAG	
P13	Fw.PsigI2.Eco	TAGAATTCCGAATTGAATGGGTTATTTGATAG	Amplification promoter of *Ct sigI2*
P14	Rv.PsigI2.Bam	TAGGATCCGGGCTTGAAAGCACAATC	
P15	Fw.PsigI3.Eco	CAGAATTCTAACCGACAAGATTTACATAAC	Amplification promoter of *Ct sigI3*
P16	Rv.PsigI3.Bam	GCGGATCCGCCTGTTCACTCCTTCAATAC	
P17	Fw.PsigI4.Eco	GCGAATTCCAAATTTTTAATGCTAAACGTCCAG	Amplification promoter of *Ct sigI4*

P18	Rv.PsigI4.Bam	GTGGATCCCAAGTTGGGTTCAAACAAATATAC	
P19	Fw.PsigI6.Eco	CTGAATTCCCATCCGATTCGCTGCAC	Amplification promoter of *Ct sigI6*
P20	Rv.PsigI6.Bam	TCGGATCCCACCCCTTATTCTTATATCGATTC	
P21	Fw.PsigI7.Eco	GAGAATTCAAATGGTATCAGCCAAAATTC	Amplification promoter of *Ct sigI7*
P22	Rv.PsigI7.Bam	GTGGATCCTCACCCAGTTGCATCAGCAAAG	
P23	Fw.PsigI8.Eco	GTGAATTCTTTCCCGTCACTTTCCGAATC	Amplification promoter of *Ct sigI8*
P24	Rv.PsigI8.Bam	GTGGATCCCCCTCCTTACTATAGAAAATTCG	

Primers used for the amplification of promoters of *C. thermocellum* DSM 1313 cellulosomal genes during the construction of pBS1ClacZ derived plasmids

#	Name	Sequence 5'→3'	Brief description
P25	Fw.Pxyn10Z.Eco	GTGAATTCGCTCAAGTAAATCAACTCCCAC	Amplification promoter of *Ct xyn10Z*
P26	Rv.Pxyn10Z.Bam	GAGGATCCGGACCTCATGTAGCAAAC	
P27	Fw.Pxyn11B.Eco	GTGAATTCTAATAAACGGCAACATAACAC	Amplification promoter of *Ct xyn11B*
P28	Rv.Pxyn11B.Bam	GCGGATCCCTTTCCCTTCGCCTGTAAATTGAC	
P29	Fw.Pce12A.Eco	CAGAATTCATGTGGTCATATTGTCAATGTAC	Amplification promoter of *Ct cel2A*
P30	Rv.Pce12A.Bam	GCGGATCCTCCCCTCCAACCTTAATATTCCAAAC	
P31	Fw.PcelE.Eco	GTGAATTCCCGGGTTTCCAACAAAGTGC	Amplification promoter of *Ct celE*
P32	Rv.PcelE.Bam	GTGGATCCCCCTTCCTATTTGGCTTG	
P33	Fw.Pcel8A.Eco	GAGAATTCGGATGAAATTTAGTCAATATTACCC	Amplification promoter of *Ct cel8A*
P34	Rv.Pcel8A.Bam	GAGGATCCCTTCAAACAGTTAATTTTCTCCC	
P35	Fw.Pcel9J.Eco	GTGAATTCGTAATTATTGCATTTTGCCCCC	Amplification promoter of *Ct cel9J*
P36	Rv.Pcel9J.Bam	GCGGATCCTGCACAATCCATACGC	
P37	Fw.Pcel9P.Eco	GTGAATTCCAATATCCACCTTACTCCGAAC	Amplification promoter of *Ct cel9P*
P38	Rv.Pcel9P.Bam	GAGGATCCCATGCTTTTACCCCTTTC	
P39	Fw.Pcel9Q.Eco	GCGAATTCAACGGGAAACAGGATTTG	Amplification promoter of *Ct cel9Q*
P40	Rv.Pcel9Q.Bam	GAGGATCCCAAATATTATTATATCATAACTCCATCTC	
P41	Fw.Pcel9U.Eco	GTGAATTCCCGTGTATTACACTTCGTATG	Amplification promoter of *Ct cel9U*
P42	Rv.Pcel9U.Bam	GCGGATCCTATTTATTATATTATTTATCTATATATATT	

		CGAAAG	
P43	Fw.Pcel9V.Eco	GTGAATTCTCCCGAAAATCTACAAATTCC	Amplification promoter of *Ct cel9V*
P44	Rv.Pcel9V.Bam	GTGGATCCCGCAAATTAACATAAACTCGC	
P45	Fw.Pcel48S.Eco	TAGAATTCGCACAAGAACTTCAAATGTTTCC	Amplification promoter of *Ct cel48*
P46	Rv.Pcel48S.Bam	TGGGATCCACGTCAAATGCAGCTGAATCAC	
P47	Fw.Pce8.Eco	GCGAATTCAAATCCCCGCTCAAATGTTG	Amplification promoter of *Ct ce8*
P48	Rv.Pce8.Bam	GCGGATCCCATGTTCTCATCTCCATTTT	
P49	Fw.PcenC.Eco	GCGAATTCAGTGTATTTATTAAATGCCTGG	Amplification promoter of *Ct cenC*
P50	Rv.PcenC.Bam	GTGGATCCCATAAGTATTAAACTATACCGTACC	
P51	Fw.PcipA.Eco	GCGAATTCATTATTTAAAAAAATGCCCCTC	Amplification promoter of *Ct cipA*
P52	Rv.PcipA.Bam	GCGGATCCCTGGGCATCTTCTTTCTTG	
P53	Fw.PcseP.Eco	GAGAATTCGTTCGCTCTACTTTAAGCCAC	Amplification promoter of *Ct cseP*
P54	Rv.PcseP.Bam	GTGGATCCTATTGCTTCCGGTTTGATTCG	
P55	Fw.Ppelb2.Eco	GCGAATTCCTCCCAATGAAATACGACCC	Amplification promoter of *Ct pelb2*
P56	Rv.Ppelb2.Bam	CAGGATCCGACACAATCATCCCTCCC	
P57	Fw.PpilZ.Eco	TGGAATTCTATTTTTATTTATCAGGTTTCAGG	Amplification promoter of *Ct pilZ*
P58	Rv.PpilZ.Bam	GAGGATCCGCAACTCAATTGCCATAAG	
P59	Fw.Ppl11.Eco	CAGAATTCATATGCCAACTGAATAATGGTGT	Amplification promoter of *Ct pl11*
P60	Rv.Ppl11.Bam	CAGGATCCAATTCACTGATATGTCCCTC	
P61	Fw.PrsgI5.Eco	GCGAATTCAATAAATAGAAATTTTATGGACC	Amplification promoter of *Ct rsgI5*
P62	Rv.PrsgI5.Bam	GCGGATCCCCGTTGCACATATTGAATATC	
P63	Fw.PrsgI9.Eco	GCGAATTCCCTGGTAGCTGAAAATCG	Amplification promoter of *Ct rsgI9*
P64	Rv.PrsgI9.Bam	GAGGATCCATCCCCCATTCACGAATAAAC	
P65	Fw.PsdbA.Eco	GTGAATTCTTAACGTCCAGGTTGCATGGC	Amplification promoter of *Ct sdbA*
P66	Rv.PsdbA.Bam	GTGGATCCCACCCTTGCTTCATATGTTTTTG	
P67	Fw.Pxgh7A.Eco	GTGAATTCTACGGGTACATCAAAGGAAAG	Amplification promoter of *Ct xgh7*
P68	Rv.Pxgh7A.Bam	GTGGATCCTAATTATGTCCCTCTTCGC	

P69	Fw.Pxyn10D.Eco	TCGAATTCAGCACGAATGCTTATATTCAAC	Amplification promoter of *Ct xynD*
P70	Rv.Pxyn10D.Bam	TCGGATCCCAACCCCTCCCCTAAAAAAG	
P71	Fw.Pxyn10Y.Eco	GTGAATTCTATGCTGTTTGCCAATATG	Amplification promoter of *Ct xyn10Y*
P72	Rv.Pxyn10Y.Bam	GTGGATCCTAAAGGGTGTTTTAAAGTTTCTTAG	
P73	Fw.Pclo1313_0563.Eco	GCGAATTCAAGGCTACAACAGAATACCG	Amplification promoter of *Ct* Clo1313_1563
P74	Rv.Pclo1313_0563.Bam	GTGGATCCGCAAATTTAATACTTCTGTTTGTG	
P75	Fw.Pclo1313_0987.Eco	GTGAATTCTGGCTTTGCATATATCCCTTG	Amplification promoter of *Ct* Clo1313_0987
P76	Rv.Pclo1313_0987.Bam	GTGGATCCTTTCTGTTCAATTTATTTGTACG	
P77	Fw.Pclo1313_1436.Eco	GTGAATTCTGAGTTGGCATGATATATGG	Amplification promoter of *Ct* Clo1313_1436
P78	Rv.Pclo1313_1436.Bam	GCGGATCCTTCCTCCCACATTGTTCCAG	
P79	Fw.Pclo1313_1494.Eco	GTGAATTCTTAGTTTTGTTCGGATTAC	Amplification promoter of *Ct* Clo1313_1494
P80	Rv.Pclo1313_1494.Bam	GAGGATCCCCCTTGATTAAATTTGCCATTTTG	
P81	Fw.Pclo1313_2216.Eco	TGGAATTCGCAGATGTAGCAGTCTATTTC	Amplification promoter of *Ct* Clo1313_2216
P82	Rv.Pclo1313_2216.Bam	GAGGATCCCCAATCCCCTCCATTATCTC	
P83	Fw.Pclo1313_2793.Eco	GTGAATTCTAAGATTGTCAGGTAATTTCTC	Amplification promoter of *Ct* Clo1313_2793
P84	Rv.Pclo1313_2793.Bam	GTGGATCCTTTGCCTCTAAGTACCTG	
P85	Fw.Pclo1313_2794.Eco	GCGAATTCATGCTAAGCCGAATGCAG	Amplification promoter of *Ct* Clo1313_2794
P86	Rv.Pclo1313_2794.Bam	GCGGATCCCCTTTATCGTAATTAATCCCTC	
P87	Fw.Pclo1313_2861.Eco	GTGAATTCCCTGTCAAATTCAAATCAAGTC	Amplification promoter of *Ct* Clo1313_2861
P88	Rv.Pclo1313_2861.Bam	GTGGATCCCTTTACTTGTTTGCCCCCTG	
P89	Fw.Pclo1313_2866.Eco	GTGAATTCGGATTGAAATGGATACAAGTC	Amplification promoter of *Ct* Clo1313_2866
P90	Rv.Pclo1313_2866.Bam	GAGGATCCACAATATCGCCTCTGTTTC	
**Primers used for site directed mutagenesis of *xyn10Z* promoter**			
**#**	**Name**	**Sequence[b] 5'→3'**	**Brief description**
P91	Fw.xynZ.short	GCGAATTCTATGTATATGATGCCGGCCGACCGACAC	Amplification of a short version of *xyn10Z* promoter
P92	Rv.xynZ.short	GCGGATCCCCTTATTTATATATTTATTGTTTCGTGAACGC	

P93	Fw.xynZ.mut1	GCGAATTCTATGTATATGATGCCGGCCGAC**G**GACACAAAAATG	Mutation from C to G in the -35 element (5' **CGAC**ac**AAA** 3' mutation in the underline nucleotide). This primer was used with Rv.xynZ.short.
P94	Fw.xynZ.mut2	GCGAATTCTATGTATATGATGCCGGCCGACC**C**ACACAAAAATG	Mutation from G to C in the -35 element. (5' **CGAC**ac**AAA** 3' mutation in the underline nucleotide). This primer was used with Rv.xynZ.short.
P95	Fw.xynZ.mut3	GCGAATTCTATGTATATGATGCCGGCCGACCG**T**CACAAAAATG	Mutation from A to T in the -35 element (5' **CGAC**ac**AAA** 3' mutation in the underline nucleotide). This primer was used with Rv.xynZ.short.
P96	Fw.xynZ.mut4	GCGAATTCTATGTATATGATGCCGGCCGACCGA**G**ACAAAAATG	Mutation from C to G in the -35 element (5' **CGAC**ac**AAA** 3' mutation in the underline nucleotides). This primer was used with Rv.xynZ.short.
P97	Fw.xynZ.mut5	GCGAATTCTATGTATATGATGCCGGCCGACCGACAC**T**AAAATG	Mutation from A to T in the -35 element (5' **CGAC**ac**AAA** 3' mutation in the underline nucleotides). This primer was used with Rv.xynZ.short.
P98	Fw.xynZ.mut6	GCGAATTCTATGTATATGATGCCGGCCGACCGACACA**T**AAATGTG	Mutation from A to T in the -35 element (5' **CGAC**ac**AAA** 3' mutation in the underline nucleotides). This primer was used with Rv.xynZ.short.
P99	Fw.xynZ.mut7	GCGAATTCTATGTATATGATGCCGGCCGACCGACACAA**T**AATGTG	Mutation from A to T in the -35 element (5' **CGAC**ac**AAA** 3' mutation in the underline nucleotides). This primer was used with Rv.xynZ.short.
P100	Rv.xynZ.mut8	GCGGATCCCCTTATTTATATATTTATTGTTTC**C**TGAACGCTC	Mutation from C to G in the -10 element (5' **CGAA**aca**AT** 3' mutation in the underline

			nucleotide). This primer was used with Fw.xynA.short.
P101	Rv.xynZ.mut9	GCGGATCCCCTTATTTATATATTTATTGTTT**G**GTGAACG CTC	Mutation from G to C in the -10 element (5' **CGAA**aca**AT** 3' mutation in the underline nucleotide). This primer was used with Fw.xynA.short.
P102	Rv.xynZ.mut10	GCGGATCCCCTTATTTATATATTTATTGTT**A**CGTGAACG CTC	Mutation from A to T in the -10 element (5' **CGAA**aca**AT** 3'mutation in the underline nucleotide). This primer was used with Fw.xynA.short.
P103	Rv.xynZ.mut11	GCGGATCCCCTTATTTATATATTTATTGT**A**TCGTGAACG CTC	Mutation from A per T in the -10 element (5' **CGAA**aca**AT** 3' mutation in the underline nucleotide). This primer was used with Fw.xynA.short.
P104	Rv.xynZ.mut12	GCGGATCCCCTTATTTATATATTT**TA**TGTTTCGTGAACG CTC	Mutation from AT per TA in the -10 element (5' **CGAA**aca**AT** 3' mutation in the underline nucleotide). This primer was used with Fw.xynA.short.
P105	Rv.xynZ.mut13	GCGGATCCCCTTATTTATATATTT**GG**TGTTTCGTGAACG CTC	Mutation from AT to CC in the -10 element (5' **CGAA**aca**AT** 3' mutation in the underline nucleotide). This primer was used with Fw.xynA.short.
**Primers used for confirmation of chromosomal integration in *B. subtilis***			
**#**	**Name**	**Sequence 5'→3'**	**Brief description**
P106<br>P107	GanQ-Cnf<br>Erm-Cnf	ATATACATTGCCCGTCGGTC<br>GCAATGAAACACGCCAAAG	Confirmation of chromosomal integration at *Bs lacA* locus. Primers binds to *ganQ* upstream of *lacA* (*ganA*), and to the *erm* gene of pAX01.
P108<br>P109	GanB-Cnf<br>XylR-Cnf	CAATGGCAGCGGCATATCC<br>GGAGCGGTTTCTATCGTTATTGATTC	Confirmation of chromosomal integration at *Bs lacA* locus. Primers binds to *ganB*

			downstream of *lacA* (*ganA*), and to the *xylR* gene of pAX01.
P110<br>P111	Ycg-Cnf<br>LacZ-Cnf	GGAAGCGTTCACAGTTTCG<br>TCCTGGAGCCCGTCAGTATC	Confirmation of chromosomal integration at *Bs amyE* locus. Primers binds to *ycgB* upstream of *amyE*, and to the *lacZ* gene of pBS1C*lacZ*.
P112<br>P113	Ldh-Cnf<br>Cat-Cnf	CAATGACCACAAGCTCATCTG<br>CTATTCAGGAATTGTCAGATAGGC	Confirmation of chromosomal integration at *Bs amyE* locus. Primers binds to *ldh* downstream of *amyE*, and to the *cat* gene of pBS1C*lacZ*.
P114<br>P115	YkoY-Cnf<br>YkrK-Cnf	CGGGCGTAATCTGAAAGACTC<br>ACAACAATTGCCAGCATAAATAAAC	Confirmation of *Bs ΔsigI-rsgI* genotype. Primers binds to *sigI-rsgI* flanking genes *ykoY* and *ykrK*.

Restriction site sequences are underlined. *Bs*, *Bacillus subtilis*; *Ct*, *Clostridium thermocellum*.

[a] These primers were designed for cloning with the In-Fusion HD Cloning Kit.

[b] The nucleotides used for site-directed mutagenesis are indicated in red and underlined.
