# Supplementary material for: Decoding Biomass-Sensing Regulons of Clostridium thermocellum Alternative Sigma-I Factors in a Heterologous Bacillus subtilis Host System
Source: PLoS One. 2016 Jan 5;11(1):e0146316. doi: 10.1371/journal.pone.0146316 (PMC4711584; doi:10.1371/journal.pone.0146316)
Supplement: S2 Table — (PDF) [file pone.0146316.s004.pdf]

S2 Table. Plasmids constructed in the present work.

Plasmid		Genotype
#	Name	
1	pLOXErysigIrsgIBs	*bla ykoY3' lox71-erm-lox66 sspD ykrK3'*
pAX01[a] derived		
2	pAX01-SigI6	*bla lacA3' xylR* P$_{xylA}$-*sigI6 erm lacA5'*
3	pAX01-SigI3	*bla lacA3' xylR* P$_{xylA}$-*sigI3 erm lacA5'*
pBS1ClacZ[b] derived		
4	pPsigI1-lacZ	*bla amyE3' cat* P$_{sigI1}$-*lacZ amyE5'*
5	pPsigI2-lacZ	*bla amyE3' cat* P$_{sigI2}$-*lacZ amyE5'*
6	pPsigI3-lacZ	*bla amyE3' cat* P$_{sigI3}$-*lacZ amyE5'*
7	pPsigI4-lacZ	*bla amyE3' cat* P$_{sigI4}$-*lacZ amyE5'*
8	pPsigI6-lacZ	*bla amyE3' cat* P$_{sigI6}$-*lacZ amyE5'*
9	pPsigI7-lacZ	*bla amyE3' cat* P$_{sigI7}$-*lacZ amyE5'*
10	pPsigI8-lacZ	*bla amyE3' cat* P$_{sigI8}$-*lacZ amyE5'*
11	pPxyn10Z-lacZ	*bla amyE3' cat* P$_{xyn10Z}$-*lacZ amyE5'*
12	pPxyn11B-lacZ	*bla amyE3' cat* P$_{xyn11B}$-*lacZ amyE5'*
13	pPce8-lacZ	*bla amyE3' cat* P$_{ce8}$-*lacZ amyE5'*
14	pPce12-lacZ	*bla amyE3' cat* P$_{cel2A}$-*lacZ amyE5'*
15	pPce1E-lacZ	*bla amyE3' cat* P$_{celE}$-*lacZ amyE5'*
16	pPcel8A-lacZ	*bla amyE3' cat* P$_{cel8A}$-*lacZ amyE5'*
17	pPcel9J-lacZ	*bla amyE3' cat* P$_{cel9J}$-*lacZ amyE5'*
18	pPcel9P-lacZ	*bla amyE3' cat* P$_{cel9P}$-*lacZ amyE5'*
19	pPcel9Q-lacZ	*bla amyE3' cat* P$_{cel9Q}$-*lacZ amyE5'*
20	pPcel9U-lacZ	*bla amyE3' cat* P$_{cel9U}$-*lacZ amyE5'*
21	pPcel9V-lacZ	*bla amyE3' cat* P$_{cel9V}$-*lacZ amyE5'*
22	pPcel48S-lacZ	*bla amyE3' cat* P$_{cel48S}$-*lacZ amyE5'*
23	pPcenC-lacZ	*bla amyE3' cat* P$_{cenC}$-*lacZ amyE5'*
24	pPcipA-lacZ	*bla amyE3' cat* P$_{cipA}$-*lacZ amyE5'*
25	pPcseP-lacZ	*bla amyE3' cat* P$_{cseP}$-*lacZ amyE5'*
26	pPpelB2-lacZ	*bla amyE3' cat* P$_{pelB2}$-*lacZ amyE5'*
27	pPpilZ-lacZ	*bla amyE3' cat* P$_{pilZ}$-*lacZ amyE5'*
28	pPpl11-lacZ	*bla amyE3' cat* P$_{pl11}$-*lacZ amyE5'*
29	pPrsgI5-lacZ	*bla amyE3' cat* P$_{rsgI5}$-*lacZ amyE5'*
30	pPrsgI9-lacZ	*bla amyE3' cat* P$_{\mathrm{rsgI9}}$-*lacZ amyE5'*
31	pPsdbA-lacZ	*bla amyE3' cat* P$_{sdbA}$-*lacZ amyE5'*
32	pPxgh74A-lacZ	*bla amyE3' cat* P$_{xgh74A}$-*lacZ amyE5'*
33	pPxyn10D-lacZ	*bla amyE3' cat* P$_{xynD}$-*lacZ amyE5'*
34	pPxyn10Y-lacZ	*bla amyE3' cat* P$_{xyn10Y}$-*lacZ amyE5'*
35	pPClo1313_0563-lacZ	*bla amyE3' cat* P$_{\mathrm{Clo1313_0563}}$-*lacZ amyE5'*
36	pPClo1313_0987-lacZ	*bla amyE3' cat* P$_{\mathrm{Clo1313_0987}}$-*lacZ amyE5'*
37	pPClo1313_1436-lacZ	*bla amyE3' cat* P$_{\mathrm{Clo1313_1436}}$-*lacZ amyE5'*
38	pPClo1313_1494-lacZ	*bla amyE3' cat* P$_{\mathrm{Clo1313_1494}}$-*lacZ amyE5'*
39	pPClo1313_2216-lacZ	*bla amyE3' cat* P$_{\mathrm{Clo1313_2216}}$-*lacZ amyE5'*
40	pPClo1313_2793-lacZ	*bla amyE3' cat* P$_{\mathrm{Clo1313_2793}}$-*lacZ amyE5'*
41	pPClo1313_2794-lacZ	*bla amyE3' cat* P$_{\mathrm{Clo1313_2794}}$-*lacZ amyE5'*
42	pPClo1313_2861-lacZ	*bla amyE3' cat* P$_{\mathrm{Clo1313_2861}}$-*lacZ amyE5'*
43	pPClo1313_2866-lacZ	*bla amyE3' cat* P$_{\mathrm{Clo1313_2866}}$-*lacZ amyE5'*
44	pPxyn10Zshort-lacZ	*bla amyE3' cat* P$_{xyn10Zshort}$-*lacZ amyE5'*

45	pPxyn10Zmut1-lacZ	*bla amyE3' cat* $P_{xyn10Zmut1}$-*lacZ amyE5'*
46	pPxyn10Zmut2-lacZ	*bla amyE3' cat* $P_{xyn10Zmut2}$-*lacZ amyE5'*
47	pPxyn10Zmut3-lacZ	*bla amyE3' cat* $P_{xyn10Zmut3}$-*lacZ amyE5'*
48	pPxyn10Zmut4-lacZ	*bla amyE3' cat* $P_{xyn10Zmut4}$-*lacZ amyE5'*
49	pPxyn10Zmut5-lacZ	*bla amyE3' cat* $P_{xyn10Zmut5}$-*lacZ amyE5'*
50	pPxyn10Zmut6-lacZ	*bla amyE3' cat* $P_{xyn10Zmut6}$-*lacZ amyE5'*
51	pPxyn10Zmut7-lacZ	*bla amyE3' cat* $P_{xyn10Zmut7}$-*lacZ amyE5'*
52	pPxyn10Zmut8-lacZ	*bla amyE3' cat* $P_{xyn10Zmut8}$-*lacZ amyE5'*
53	pPxyn10Zmut9-lacZ	*bla amyE3' cat* $P_{xyn10Zmut9}$-*lacZ amyE5'*
54	pPxyn10Zmut10-lacZ	*bla amyE3' cat* $P_{xyn10Zmut10}$-*lacZ amyE5'*
55	pPxyn10Zmut11-lacZ	*bla amyE3' cat* $P_{xyn10Zmut11}$-*lacZ amyE5'*
56	pPxyn10Zmut12-lacZ	*bla amyE3' cat* $P_{xyn10Zmut12}$-*lacZ amyE5'*
57	pPxyn10Zmut13-lacZ	*bla amyE3' cat* $P_{xyn10Zmut13}$-*lacZ amyE5'*

[a] This plasmid was developed by Härtl and co-workers [28] and donated by Dr. Avigdor Eldar, Department of Molecular Microbiology and Biotechnology, Tel Aviv University.

[b] This plasmid was developed by Radeck and co-workers [29] and obtained from the Bacillus Genetic Stock Center.
