# Supplementary material for: Decoding Biomass-Sensing Regulons of Clostridium thermocellum Alternative Sigma-I Factors in a Heterologous Bacillus subtilis Host System
Source: PLoS One. 2016 Jan 5;11(1):e0146316. doi: 10.1371/journal.pone.0146316 (PMC4711584; doi:10.1371/journal.pone.0146316)
Supplement: S3 Table — (PDF) [file pone.0146316.s005.pdf]

S3 Table. *Bacillus subtilis* strains constructed in the present work.

Strain		Relevant genotype
#	Name	
1	PY79[a]	Protrophic derivative of *B. subtilis* 168
2	BKE13460[b]	*B. subtilis* 168 *ΔrsgI::loxP-erm-loxP*
3	CO01	PY79 Δ(*sigI-rsgI*)::*loxP-erm-loxP*
4	CO02	PY79 Δ(*sigI-rsgI*)
pBSClacZ derived strains		
5	LO001	CO02 *amyE*::(P_{sigI1}-*lacZ cat*)
6	LO002	CO02 *amyE*::(P_{sigI2}-*lacZ cat*)
7	LO003	CO02 *amyE*::(P_{sigI3}-*lacZ cat*)
8	LO004	CO02 *amyE*::(P_{sigI4}-*lacZ cat*)
9	LO005	CO02 *amyE*::(P_{sigI6}-*lacZ cat*)
10	LO006	CO02 *amyE*::(P_{sigI7}-*lacZ cat*)
11	LO007	CO02 *amyE*::(P_{sigI8}-*lacZ cat*)
12	LO008	CO02 *amyE*::(P_{xyn10Z}-*lacZ cat*)
13	LO009	CO02 *amyE*::(P_{xyn11B}-*lacZ cat*)
14	LO010	CO02 *amyE*::(P_{ce8}-*lacZ cat*)
15	LO011	CO02 *amyE*::(P_{ce12}-*lacZ cat*)
16	LO012	CO02 *amyE*::(P_{celE}-*lacZ cat*)
17	LO013	CO02 *amyE*::(P_{cel8A}-*lacZ cat*)
18	LO014	CO02 *amyE*::(P_{cel9J}-*lacZ cat*)
19	LO015	CO02 *amyE*::(P_{celP}-*lacZ cat*)
20	LO016	CO02 *amyE*::(P_{cel9Q}-*lacZ cat*)
21	LO017	CO02 *amyE*::(P_{cel9U}-*lacZ cat*)
22	LO018	CO02 *amyE*::(P_{cel9V}-*lacZ cat*)
23	LO019	CO02 *amyE*::(P_{cel48S}-*lacZ cat*)
24	LO020	CO02 *amyE*::(P_{cenC}-*lacZ cat*)
25	LO021	CO02 *amyE*::(P_{cipA}-*lacZ cat*)
26	LO022	CO02 *amyE*::(P_{cesP}-*lacZ cat*)
27	LO023	CO02 *amyE*::(P_{pelB2}-*lacZ cat*)
28	LO024	CO02 *amyE*::(P_{pilZ}-*lacZ cat*)
29	LO025	CO02 *amyE*::(P_{pl11}-*lacZ cat*)
30	LO026	CO02 *amyE*::(P_{rsgI5}-*lacZ cat*)
31	LO027	CO02 *amyE*::(P_{rsgI9}-*lacZ cat*)
32	LO028	CO02 *amyE*::(P_{sdbA}-*lacZ cat*)
33	LO029	CO02 *amyE*::(P_{xgh74A}-*lacZ cat*)
34	LO030	CO02 *amyE*::(P_{xyn10D}-*lacZ cat*)
35	LO031	CO02 *amyE*::(P_{xyn10Y}-*lacZ cat*)
36	LO032	CO02 *amyE*::($P_{Clo1313\_0563}$-*lacZ cat*)
37	LO033	CO02 *amyE*::($P_{Clo1313\_0987}$-*lacZ cat*)
38	LO034	CO02 *amyE*::($P_{Clo1313\_1436}$-*lacZ cat*)
39	LO035	CO02 *amyE*::($P_{Clo1313\_1494}$-*lacZ cat*)
40	LO036	CO02 *amyE*::($P_{Clo1313\_2216}$-*lacZ cat*)
41	LO037	CO02 *amyE*::($P_{Clo1313\_2793}$-*lacZ cat*)
42	LO038	CO02 *amyE*::($P_{Clo1313\_2794}$-*lacZ cat*)
43	LO039	CO02 *amyE*::($P_{Clo1313\_2861}$-*lacZ cat*)
44	LO040	CO02 *amyE*::($P_{Clo1313\_2866}$-*lacZ cat*)
45	LO041	CO02 *amyE*::($P_{xyn10Zshort}$-*lacZ cat*)

46	LO042	CO02 *amyE*::($P_{xyn10Zmut1}$-*lacZ cat*)
47	LO043	CO02 *amyE*::($P_{xyn10Zmut2}$-*lacZ cat*)
48	LO044	CO02 *amyE*::($P_{xyn10Zmut3}$-*lacZ cat*)
49	LO045	CO02 *amyE*::($P_{xyn10Zmut4}$-*lacZ cat*)
50	LO046	CO02 *amyE*::($P_{xyn10Zmut5}$-*lacZ cat*)
51	LO047	CO02 *amyE*::($P_{xyn10Zmut6}$-*lacZ cat*)
52	LO048	CO02 *amyE*::($P_{xyn10Zmut7}$-*lacZ cat*)
53	LO049	CO02 *amyE*::($P_{xyn10Zmut8}$-*lacZ cat*)
54	LO050	CO02 *amyE*::($P_{xyn10Zmut9}$-*lacZ cat*)
55	LO051	CO02 *amyE*::($P_{xyn10Zmut10}$-*lacZ cat*)
56	LO052	CO02 *amyE*::($P_{xyn10Zmut11}$-*lacZ cat*)
57	LO053	CO02 *amyE*::($P_{xyn10Zmut12}$-*lacZ cat*)
58	LO054	CO02 *amyE*::($P_{xyn10Zmut13}$-*lacZ cat*)
pAX01-SigI6 derived strains		
59	LO601	CO02 *lacA*::(P_{xyl}-*sigI6 erm*) *amyE::*(P_{sigI1}-*lacZ cat*)
60	LO602	CO02 *lacA*::(P_{xyl}-*sigI6 erm*) *amyE::*(P_{sigI2}-*lacZ cat*)
61	LO603	CO02 *lacA*::(P_{xyl}-*sigI6 erm*) *amyE::*(P_{sigI3}-*lacZ cat*)
62	LO604	CO02 *lacA*::(P_{xyl}-*sigI6 erm*) *amyE*::(P_{sigI4}-*lacZ cat*)
63	LO605	CO02 *lacA*::(P_{xyl}-*sigI6 erm*) *amyE::*(P_{sigI6}-*lacZ cat*)
64	LO606	CO02 *lacA*::(P_{xyl}-*sigI6 erm*) *amyE*::(P_{sigI7}-*lacZ cat*)
65	LO607	CO02 *lacA*::(P_{xyl}-*sigI6 erm*) *amyE*::(P_{sigI8}-*lacZ cat*)
66	LO608	CO02 *lacA*::(P_{xyl}-*sigI6 erm*) *amyE*::(P_{xyn10Z}-*lacZ cat*)
67	LO609	CO02 *lacA*::(P_{xyl}-*sigI6 erm*) *amyE*::(P_{xyn11B}-*lacZ cat*)
68	LO610	CO02 *lacA*::(P_{xyl}-*sigI6 erm*) *amyE*::(P_{ce8}-*lacZ cat*)
69	LO611	CO02 *lacA*::(P_{xyl}-*sigI6 erm*) *amyE*::(P_{ce12}-*lacZ cat*)
70	LO612	CO02 *lacA*::(P_{xyl}-*sigI6 erm*) *amyE*::(P_{celE}-*lacZ cat*)
71	LO613	CO02 *lacA*::(P_{xyl}-*sigI6 erm*) *amyE*::(P_{cel8A}-*lacZ cat*)
72	LO614	CO02 *lacA*::(P_{xyl}-*sigI6 erm*) *amyE*::(P_{cel9J}-*lacZ cat*)
73	LO615	CO02 *lacA*::(P_{xyl}-*sigI6 erm*) *amyE*::(P_{celP}-*lacZ cat*)
74	LO616	CO02 *lacA*::(P_{xyl}-*sigI6 erm*) *amyE*::(P_{cel9Q}-*lacZ cat*)
75	LO617	CO02 *lacA*::(P_{xyl}-*sigI6 erm*) *amyE*::(P_{cel9U}-*lacZ cat*)
76	LO618	CO02 *lacA*::(P_{xyl}-*sigI6 erm*) *amyE*::(P_{cel9V}-*lacZ cat*)
77	LO619	CO02 *lacA*::(P_{xyl}-*sigI6 erm*) *amyE::*(P_{cel48S}-*lacZ cat*)
78	LO620	CO02 *lacA*::(P_{xyl}-*sigI6 erm*) *amyE*::(P_{cenC}-*lacZ cat*)
79	LO621	CO02 *lacA*::(P_{xyl}-*sigI6 erm*) *amyE::*(P_{cipA}-*lacZ cat*)
80	LO622	CO02 *lacA*::(P_{xyl}-*sigI6 erm*) *amyE*::(P_{cesP}-*lacZ cat*)
81	LO623	CO02 *lacA*::(P_{xyl}-*sigI6 erm*) *amyE*::(P_{pelB2}-*lacZ cat*)
82	LO624	CO02 *lacA*::(P_{xyl}-*sigI6 erm*) *amyE*::(P_{pilZ}-*lacZ cat*)
83	LO625	CO02 *lacA*::(P_{xyl}-*sigI6 erm*) *amyE*::(P_{pl11}-*lacZ cat*)
84	LO626	CO02 *lacA*::(P_{xyl}-*sigI6 erm*) *amyE*::(P_{rsgI5}-*lacZ cat*)
85	LO627	CO02 *lacA*::(P_{xyl}-*sigI6 erm*) *amyE*::(P_{rsgI9}-*lacZ cat*)
86	LO628	CO02 *lacA*::(P_{xyl}-*sigI6 erm*) *amyE*::(P_{sdbA}-*lacZ cat*)
87	LO629	CO02 *lacA*::(P_{xyl}-*sigI6 erm*) *amyE*::(P_{xgh74A}-*lacZ cat*)
88	LO630	CO02 *lacA*::(P_{xyl}-*sigI6 erm*) *amyE::(*P_{xyn10D}-*lacZ cat*)
89	LO631	CO02 *lacA*::(P_{xyl}-*sigI6 erm*) *amyE*::(P_{xyn10Y}-*lacZ cat*)
90	LO632	CO02 *lacA*::(P_{xyl}-*sigI6 erm*) *amyE*::($P_{\mathrm{Clo1313\_0563}}$-*lacZ cat*)
91	LO633	CO02 *lacA*::(P_{xyl}-*sigI6 erm*) *amyE*::($P_{\mathrm{Clo1313\_0987}}$-*lacZ cat*)
92	LO634	CO02 *lacA*::(P_{xyl}-*sigI6 erm*) *amyE*::($P_{\mathrm{Clo1313\_1436}}$-*lacZ cat*)
93	LO635	CO02 *lacA*::(P_{xyl}-*sigI6 erm*) *amyE*::($P_{\mathrm{Clo1313\_1494}}$-*lacZ cat*)

94	LO636	CO02 *lacA*::(P_{xyl}-*sigI6 erm*) *amyE*::($P_{Clo1313\_2216}$-*lacZ cat*)
95	LO637	CO02 *lacA*::(P_{xyl}-*sigI6 erm*) *amyE*::($P_{Clo1313\_2793}$-*lacZ cat*)
96	LO638	CO02 *lacA*::(P_{xyl}-*sigI6 erm*) *amyE*::($P_{Clo1313\_2794}$-*lacZ cat*)
97	LO639	CO02 *lacA*::(P_{xyl}-*sigI6 erm*) *amyE*::($P_{Clo1313\_2861}$-*lacZ cat*)
98	LO640	CO02 *lacA*::(P_{xyl}-*sigI6 erm*) *amyE*::($P_{Clo1313\_2866}$-*lacZ cat*)
99	LO641	CO02 *lacA*::(P_{xyl}-*sigI6 erm*) *amyE*::($P_{xyn10Zshort}$-*lacZ cat*)
100	LO642	CO02 *lacA*::(P_{xyl}-*sigI6 erm*) *amyE*::($P_{xyn10Zmut1}$-*lacZ cat*)
101	LO643	CO02 *lacA*::(P_{xyl}-*sigI6 erm*) *amyE*::($P_{xyn10Zmut2}$-*lacZ cat*)
102	LO644	CO02 *lacA*::(P_{xyl}-*sigI6 erm*) *amyE*::($P_{xyn10Zmut3}$-*lacZ cat*)
103	LO645	CO02 *lacA*::(P_{xyl}-*sigI6 erm*) *amyE*::($P_{xyn10Zmut4}$-*lacZ cat*)
104	LO646	CO02 *lacA*::(P_{xyl}-*sigI6 erm*) *amyE*::($P_{xyn10Zmut5}$-*lacZ cat*)
105	LO647	CO02 *lacA*::(P_{xyl}-*sigI6 erm*) *amyE*::($P_{xyn10Zmut6}$-*lacZ cat*)
106	LO648	CO02 *lacA*::(P_{xyl}-*sigI6 erm*) *amyE*::($P_{xyn10Zmut7}$-*lacZ cat*)
107	LO649	CO02 *lacA*::(P_{xyl}-*sigI6 erm*) *amyE*::($P_{xyn10Zmut8}$-*lacZ cat*)
108	LO650	CO02 *lacA*::(P_{xyl}-*sigI6 erm*) *amyE*::($P_{xyn10Zmut9}$-*lacZ cat*)
109	LO651	CO02 *lacA*::(P_{xyl}-*sigI6 erm*) *amyE*::($P_{xyn10Zmut10}$-*lacZ cat*)
110	LO652	CO02 *lacA*::(P_{xyl}-*sigI6 erm*) *amyE*::($P_{xyn10Zmut11}$-*lacZ cat*)
111	LO653	CO02 *lacA*::(P_{xyl}-*sigI6 erm*) *amyE*::($P_{xyn10Zmut12}$-*lacZ cat*)
112	LO654	CO02 *lacA*::(P_{xyl}-*sigI6 erm*) *amyE*::($P_{xyn10Zmut13}$-*lacZ cat*)
pAX01-SigI3 derived strains		
113	L0301	CO02 *lacA*::(P_{xyl}-*sigI3 erm*) *amyE::*(P_{sigI1}-*lacZ cat*)
114	L0302	CO02 *lacA*::(P_{xyl}-*sigI3 erm*) *amyE::*(P_{sigI2}-*lacZ cat*)
115	L0303	CO02 *lacA*::(P_{xyl}-*sigI3 erm*) *amyE::*(P_{sigI3}-*lacZ cat*)
116	L0304	CO02 *lacA*::(P_{xyl}-*sigI3 erm*) *amyE*::(P_{sigI4}-*lacZ cat*)
117	L0305	CO02 *lacA*::(P_{xyl}-*sigI3 erm*) *amyE::*(P_{sigI6}-*lacZ cat*)
118	L0306	CO02 *lacA*::(P_{xyl}-*sigI3 erm*) *amyE*::(P_{sigI7}-*lacZ cat*)
119	L0307	CO02 *lacA*::(P_{xyl}-*sigI3 erm*) *amyE*::(P_{sigI8}-*lacZ cat*)
120	L0308	CO02 *lacA*::(P_{xyl}-*sigI3 erm*) *amyE*::(P_{xyn10Z}-*lacZ cat*)
121	L0309	CO02 *lacA*::(P_{xyl}-*sigI3 erm*) *amyE*::(P_{xyn11B}-*lacZ cat*)
122	L0310	CO02 *lacA*::(P_{xyl}-*sigI3 erm*) *amyE*::(P_{ce8}-*lacZ cat*)
123	L0311	CO02 *lacA*::(P_{xyl}-*sigI3 erm*) *amyE*::(P_{ce12}-*lacZ cat*)
124	L0312	CO02 *lacA*::(P_{xyl}-*sigI3 erm*) *amyE*::(P_{celE}-*lacZ cat*)
125	L0313	CO02 *lacA*::(P_{xyl}-*sigI3 erm*) *amyE*::(P_{cel8A}-*lacZ cat*)
126	L0314	CO02 *lacA*::(P_{xyl}-*sigI3 erm*) *amyE*::(P_{cel9J}-*lacZ cat*)
127	L0315	CO02 *lacA*::(P_{xyl}-*sigI3 erm*) *amyE*::(P_{celP}-*lacZ cat*)
128	L0316	CO02 *lacA*::(P_{xyl}-*sigI3 erm*) *amyE*::(P_{cel9Q}-*lacZ cat*)
129	L0317	CO02 *lacA*::(P_{xyl}-*sigI3 erm*) *amyE*::(P_{cel9U}-*lacZ cat*)
130	L0318	CO02 *lacA*::(P_{xyl}-*sigI3 erm*) *amyE*::(P_{cel9V}-*lacZ cat*)
131	L0319	CO02 *lacA*::(P_{xyl}-*sigI3 erm*) *amyE::*(P_{cel48S}-*lacZ cat*)
132	L0320	CO02 *lacA*::(P_{xyl}-*sigI3 erm*) *amyE*::(P_{cenC}-*lacZ cat*)
133	L0321	CO02 *lacA*::(P_{xyl}-*sigI3 erm*) *amyE::*(P_{cipA}-*lacZ cat*)
134	L0322	CO02 *lacA*::(P_{xyl}-*sigI3 erm*) *amyE*::(P_{cesP}-*lacZ cat*)
135	L0323	CO02 *lacA*::(P_{xyl}-*sigI3 erm*) *amyE*::(P_{pelB2}-*lacZ cat*)
136	L0324	CO02 *lacA*::(P_{xyl}-*sigI3 erm*) *amyE*::(P_{pilZ}-*lacZ cat*)
137	L0325	CO02 *lacA*::(P_{xyl}-*sigI3 erm*) *amyE*::(P_{pl11}-*lacZ cat*)
138	L0326	CO02 *lacA*::(P_{xyl}-*sigI3 erm*) *amyE*::(P_{rsgI5}-*lacZ cat*)
139	L0327	CO02 *lacA*::(P_{xyl}-*sigI3 erm*) *amyE*::(P_{rsgI9}-*lacZ cat*)
140	L0328	CO02 *lacA*::(P_{xyl}-*sigI3 erm*) *amyE*::(P_{sdbA}-*lacZ cat*)
141	L0329	CO02 *lacA*::(P_{xyl}-*sigI3 erm*) *amyE*::(P_{xgh74A}-*lacZ cat*)

142	L0330	CO02 *lacA*::(P_{xyl}-*sigI3 erm*) *amyE::(*P_{xyn10D}-*lacZ cat*)
143	L0331	CO02 *lacA*::(P_{xyl}-*sigI3 erm*) *amyE*::(P_{xyn10Y}-*lacZ cat*)
144	L0332	CO02 *lacA*::(P_{xyl}-*sigI3 erm*) *amyE*::($P_{Clo1313\_0563}$-*lacZ cat*)
145	L0333	CO02 *lacA*::(P_{xyl}-*sigI3 erm*) *amyE*::($P_{Clo1313\_0987}$-*lacZ cat*)
146	L0334	CO02 *lacA*::(P_{xyl}-*sigI3 erm*) *amyE*::($P_{Clo1313\_1436}$-*lacZ cat*)
147	L0335	CO02 *lacA*::(P_{xyl}-*sigI3 erm*) *amyE*::($P_{Clo1313\_1494}$-*lacZ cat*)
148	L0336	CO02 *lacA*::(P_{xyl}-*sigI3 erm*) *amyE*::($P_{Clo1313\_2216}$-*lacZ cat*)
149	L0337	CO02 *lacA*::(P_{xyl}-*sigI3 erm*) *amyE*::($P_{Clo1313\_2793}$-*lacZ cat*)
150	L0338	CO02 *lacA*::(P_{xyl}-*sigI3 erm*) *amyE*::($P_{Clo1313\_2794}$-*lacZ cat*)
151	L0339	CO02 *lacA*::(P_{xyl}-*sigI3 erm*) *amyE*::($P_{Clo1313\_2861}$-*lacZ cat*)
152	L0340	CO02 *lacA*::(P_{xyl}-*sigI3 erm*) *amyE*::($P_{Clo1313\_2866}$-*lacZ cat*)
153	L0341	CO02 *lacA*::(P_{xyl}-*sigI3 erm*) *amyE*::($P_{xyn10Zshort}$-*lacZ cat*)
154	L0342	CO02 *lacA*::(P_{xyl}-*sigI3 erm*) *amyE*::($P_{xyn10Zmut1}$-*lacZ cat*)
155	L0343	CO02 *lacA*::(P_{xyl}-*sigI3 erm*) *amyE*::($P_{xyn10Zmut2}$-*lacZ cat*)
156	L0344	CO02 *lacA*::(P_{xyl}-*sigI3 erm*) *amyE*::($P_{xyn10Zmut3}$-*lacZ cat*)
157	L0345	CO02 *lacA*::(P_{xyl}-*sigI3 erm*) *amyE*::($P_{xyn10Zmut4}$-*lacZ cat*)
158	L0346	CO02 *lacA*::(P_{xyl}-*sigI3 erm*) *amyE*::($P_{xyn10Zmut5}$-*lacZ cat*)
159	L0347	CO02 *lacA*::(P_{xyl}-*sigI3 erm*) *amyE*::($P_{xyn10Zmut6}$-*lacZ cat*)
160	L0348	CO02 *lacA*::(P_{xyl}-*sigI3 erm*) *amyE*::($P_{xyn10Zmut7}$-*lacZ cat*)
161	L0349	CO02 *lacA*::(P_{xyl}-*sigI3 erm*) *amyE*::($P_{xyn10Zmut8}$-*lacZ cat*)
162	L0350	CO02 *lacA*::(P_{xyl}-*sigI3 erm*) *amyE*::($P_{xyn10Zmut9}$-*lacZ cat*)
163	L0351	CO02 *lacA*::(P_{xyl}-*sigI3 erm*) *amyE*::($P_{xyn10Zmut10}$-*lacZ cat*)
164	L0352	CO02 *lacA*::(P_{xyl}-*sigI3 erm*) *amyE*::($P_{xyn10Zmut11}$-*lacZ cat*)
165	L0353	CO02 *lacA*::(P_{xyl}-*sigI3 erm*) *amyE*::($P_{xyn10Zmut12}$-*lacZ cat*)
166	L0354	CO02 *lacA*::(P_{xyl}-*sigI3 erm*) *amyE*::($P_{xyn10Zmut13}$-*lacZ cat*)

[a] Strain donated by Dr. Avigdor Eldar, Department of Molecular Microbiology and Biotechnology, Tel Aviv University.

[b] Strain obtained from the Bacillus Genetic Stock Center (BGSC).
