# Supplementary material for: Decoding Biomass-Sensing Regulons of Clostridium thermocellum Alternative Sigma-I Factors in a Heterologous Bacillus subtilis Host System
Source: PLoS One. 2016 Jan 5;11(1):e0146316. doi: 10.1371/journal.pone.0146316 (PMC4711584; doi:10.1371/journal.pone.0146316)
Supplement: S4 Table — (PDF) [file pone.0146316.s006.pdf]

S4 Table. Alignment of experimentally confirmed and putative *sigI* promoters from different *Bacillales* species, including the experimentally confirmed *sigI*-dependent promoters of the *B. subtilis bcrC* and *mreBH* genes.

Species	Promoter sequences (5'→3')
B. subtilis sigI[a,b]	aa**ACCCCC**tt**AA**ttctttagaaaggca**CGAA**atca**T**
B. subtilis bcrC[b]	atc**CCCCC**ag**AA**accgcgattcctctt**CGAA**ttctc
B. subtilis mreBH[b]	ac**ACCCCC**aa**AA**atcgcagtatttctga**GAA**actt**T**
B. licheniformis ATCC 14580[b]	aa**ACCCCC**tt**AA**tcgtcaaacagatca**CGAA**ttgt**T**
B. thuringiensis sv. Israelensis ATCC 35646[b]	tg**ACCCCC**at**AA**aactatgtattcctc**CGAA**tatg**T**
Bacillus sp. NRRL B-14911[b]	cc**ACCCCC**aa**AA**gctcctctttccggg**CGAA**gctt**T**
Bacillus tequilensis	aa**ACCCCC**tt**AA**ttctttagaaagaca**CGAA**atca**T**
Bacillus sp TH008	aa**ACCCCC**ct**AA**tcctcaaagatatat**CGAA**ttga**T**
Bacillus vallismortis	aa**ACCCCC**tt**AA**ttctctagaaaggca**CGAA**atca**T**
Bacillus sonorensis	aa**ACCCCC**ct**AA**tcaacaagtaaataa**CGAA**ttga**T**
Geobacillus thermoglucosidasus	tg**ACC**aa**C**aa**AA**atccgctatgatgta**CGAA**tgata
Bacillus sp NSP91	aa**ACCCCC**tt**AA**tcgtcaaagaaacaa**CGAA**ttgt**T**
Bacillus mojavensis	aa**ACCCCC**aa**AA**ttctctttaagggca**CGAA**atcc**T**
Bacillus stratophericus LAMA 587	at**ACCCCC**tt**A**tttctttgcttttaaa**CGAA**acgt**T**
Bacillus xiamenensis	at**ACCCCC**tt**A**tttctttgcttttaaa**CGAA**acgt**T**
Salinibacillus aidingensis MSP4	aa**ACCCCC**aa**AA**ttctctttaaaggca**CGAA**atcc**T**
Bacillus amyloliquefaciens FZB42	aa**ACCCCC**tt**AA**ttctgcatgggggca**CGAA**atca**T**
Bacillus pumilus ATCC 7061	at**ACCCCC**tt**A**tttctttgcatttaga**CGAA**acac**T**
Bacillus mycoides Rock1-4	tg**ACCCCC**at**AA**aatttttatttcctt**CGAA**tatg**T**
Bacillus pseudomycoides DSM 12442	tg**ACCCCC**at**AA**aatttttatttcctc**CGAA**tatg**T**
Bacillus weihenstephanensis KBAB4	tg**ACCCCC**at**AA**aattatttgttcctt**CGAA**tata**T**
Bacillus cereus ATCC 10987	tg**ACCCCC**at**AA**aacgatgtattcctc**CGAA**tata**T**
Bacillus anthracis strain Ames	cg**ACCCCC**at**AA**aactttgtattcctc**CGAA**tatg**T**
Bacillus thuringiensis subsp. konkukian	tg**ACCCCC**at**AA**aactttgtattcctc**CGAA**tatg**T**
Bacillus sp. SG-1	gt**ACCCCC**ac**AA**gctattggaagttct**CG**t**A**actt**T**

Bacillus coahuilensis m4-4	tt**ACCCCC**ta**AA**actaattttatcatc**CG**t**A**tgtta
Geobacillus kaustophilus HTA426	aat**CCCC**tttg**A**acgacggtcatatttg**GA**ttgcga
Geobacillus stearothermophilus NUB3621	ta**ACC**a**CC**ag**A**tttccgttaagatgta**CGAA**tgata
Consensus	**ACCCCC--AA** 15(N) **CGAA----T**

The most conserved bases are shown in bold capital fonts. Underlined are the -35 and -10 promoter elements proposed by Tseng and Shaw [34].

[a] Promoter sequence confirmed experimentally by Asai and co-workers [17].

[b] Promoter sequence confirmed experimentally by Tseng and Shaw [34].
