# Supplementary material for: Decoding Biomass-Sensing Regulons of Clostridium thermocellum Alternative Sigma-I Factors in a Heterologous Bacillus subtilis Host System
Source: PLoS One. 2016 Jan 5;11(1):e0146316. doi: 10.1371/journal.pone.0146316 (PMC4711584; doi:10.1371/journal.pone.0146316)
Supplement: S5 Table — (PDF) [file pone.0146316.s007.pdf]

S5 Table. Alignments of predicted *sigI* promoters from *Clostridium clariflavum*, *Acetivibrio cellulolyticus*, *Pseudobacteroides cellulosolvens*, *Clostridium thermocellum* and *Clostridium straminisolvens*

Species	Locus tag *sigI-rsgI*[a] or Genbank accession number	RsgI C-terminal domain	Promoter region 5'→3'	5' UTR
C. clariflavum	(Clocl_1054-1053)	CBM3	aaccccccta**AAA**aatcaacttactta**CGaA**tttctg	269
C. clariflavum	(Clocl_2045-2044)	UNK	taccgacaa**AAA**aat-agttcaatta**CGaA**tgaact	18
C. clariflavum	(Clocl_2099-2098)	CBM42	tccgaacat**AAA**agaaaaaacgattt**CGaA**tatata	173
C. clariflavum	(Clocl_2748-2747)	2xPA14	atgcgaaat**AAA**aaatagaataagga**CGaA**tatact	478
C. clariflavum	(Clocl_2798-2797)	Trypan_PARP	ttgcgaatg**AAA**ataaggaaagtaaa**CGaA**ttaact	25
C. clariflavum	(Clocl_2844-2843)	CBM3	tgtgcacta**AAA**tgt-gatttgttgt**CGaA**ttagta	20
C. clariflavum	(Clocl_4009-4008)	CBM3	gtggatacc**AAA**tta-tttatttttg**CGaA**ttttta	19
A. cellulolyticus	WP_010243057.1-WP_010243060.1	CBM42	tcccaactt**AAA**aatagaattactca**CGaA**ataata	145
A. cellulolyticus	WP_010246332.1-WP_010246331.1	PA14-CBM35	gtatcccta**AAA**aaagtttgtgttta**CGcA**ttaaag	14
A. cellulolyticus	WP_010681061.1-WP_010681060.1	CBM3	atgcacact**AAA**tta-attattttt**CGaA**ttttat	20
A. cellulolyticus	WP_010247803.1-WP_010247800.1	2xPA14	attcccgac**AAA**aaatatttttcaa**CGaA**ttttat	108
A. cellulolyticus	WP_010247809.1-WP_010247806.1	Protease	atatacact**AAA**taaatataatccta**CGt**tattata	18
A. cellulolyticus	WP_040428536.1-WP_010248061.1	CBM3	tgtgcacta**AAA**taatgagcagttat**CGaA**ttaata	41
A. cellulolyticus	WP_040428617.1-WP_010248923.1	CBM3	cctatacag**AAA**atttagtgtattat**CGaA**atatat	16
A. cellulolyticus	WP_010250840.1-WP_010250842.1	UNK	taacgaata**AAA**aatgattcaatatt**CGaA**tgtagc	16
A. cellulolyticus	WP_010251306.1-WP_010251305.1	CBM3	aaccccta**AAA**tttattgtaagta**CGaA**ttttta	162
A. cellulolyticus	WP_010252667.1-WP_010252672.1	UNK	taccgtcaa**AAA**aagttacttggttt**CGtA**taacta	285
P. cellulosolvens	(Bccel_0204-0205)	2xPA14	aaacgacct**AAA**aat-tattttcatt**CGaA**tattta	240
P. cellulosolvens	(Bccel_0630-0629)	FN3	ctgctctta**AAA**atcatacttcttat**CGaA**caatat	15
P. cellulosolvens	(Bccel_0725-0726)	CBM3	cctatactg**AAA**aatttgaattttat**CGaA**atatat	16
P. cellulosolvens	(Bccel_3092-3091)	CBM42	tccgaactc**AAA**aataaattcgatta**CGaA**ttaatc	184
P. cellulosolvens	(Bccel_3399-3398)	UNK	ccccgtcac**AAA**atttactggttcatg**GaA**accata	15

P. cellulosolvens	(Bccel_5133-5134)	UNK	agccacata**AAA**atattttatactta**CG**cctttatt	14
P. cellulosolvens	(Bccel_5156-5155)	UNK	gaccgaaca**AAA**acaagcaaaaattt**CGaA**tatact	19
P. cellulosolvens	(Bccel_5622-5623)	PA14-CBM35	tatatcccg**AAA**aagttttaaatgca**CG**c**A**tatata	15
P. cellulosolvens	(Bccel_5637-5636)	CBM3	agccacatc**AAA**aatttactttactc**CGaA**acacta	22
C. thermocellum	(Clo1313_0104-0105)	UNK	cgactgatgtt**A**tt-taaatttgtgt**CGaA**ctttgc	54
C. thermocellum	(Clo1313_0525-0524)	UNK	ttccgaatc**AAA**atgaaatccatata**CGaA**ttttct	18
C. thermocellum	(Clo1313_1818-1817)	CBM3	gtccagctg**AAA**attttctgccacgc**CG**c**A**ttaatt	15
C. thermocellum	(Clo1313_1911-1910)	2xPA14	gaacccctc**AAA**aaaatcatttggtg**CGtA**caagta	15
C. thermocellum	(Clo1313_1961-1962)	CBM3	tatcccccg**AAA**aaatgttcccttta**CGaA**ataact	149
C. thermocellum	(Clo1313_2174-2173)	CBM3	tatacacaa**AAA**aa-gcagatgtata**CGaA**gtaatc	18
C. thermocellum	(Clo1313_2778-2777)	GH10	atgcgacat**AAA**accattccggtata**CGaA**tcgata	22
C. straminisolvens	GAE88373.1-GAE88374.1	CBM3	gtccaacta**AAA**gtttgttgccacat**CG**c**A**ttaatc	15
C. straminisolvens	GAE88571.1-GAE88572.1	CBM3	tatacataa**AAA**aa-gcaggcttgaa**CGaA**gtaatc	19
C. straminisolvens	GAE88883.1-GAE88884.1	2xPA14	aaacccctc**AAA**aaa-taactttgtg**CGtA**caagta	17
C. straminisolvens	GAE89281.1-GAE89280.1	CBM3	tatcccct**AAA**atttgttcctctta**CGaA**ataact	161
C. straminisolvens	GAE90135.1-GAE90134.1	UNK	cgactagtggtttg-tagatttatgt**CGaA**ctttgc	63
C. straminisolvens	GAE90430.1-GAE90432.1	UNK	taccgaatt**AAA**atagaagtcatata**CGaA**tcctct	20
C. straminisolvens	GAE90492.1-GAE90491.1	GH10	atgcgacat**AAA**gctattccagtcta**CGaA**ttcata	24
Consensus			**AAA 14-13(N) CGWA**	
Bacillales consensus			**ACCCCC--AA 15(N) CGAA----T**	

The most conserved bases are shown in bold capital fonts. The sequences of *C. thermocellum* and *C. straminisolvens* are the same sequences shown in Table 1 and were included for comparison. W represents A or T.

[a] Clocl, Bccel and Clo1313 are the locus tag prefixes of *C. clariflavum*, *P. cellulosolvens* and *C. thermocellum* DSM 1313, respectively.
