# Supplementary material for: Decoding Biomass-Sensing Regulons of Clostridium thermocellum Alternative Sigma-I Factors in a Heterologous Bacillus subtilis Host System
Source: PLoS One. 2016 Jan 5;11(1):e0146316. doi: 10.1371/journal.pone.0146316 (PMC4711584; doi:10.1371/journal.pone.0146316)
Supplement: S6 Table — (PDF) [file pone.0146316.s008.pdf]

**S6 Table. σ$^{I6}$-dependent promoter sequence alignment of *xyn11B-xyn11A* operon and *xyn11A* of available sequences of *C. thermocellum* and C. *straminisolvens* JCM 21531.**

| Species | Gene (s) | Tag locus[a] or Genbank accession number | Promoter region 5'→3' | | | 5' UTR |
|---|---|---|---|---|---|---|
| *Ct* DSM 1313 | *xyn11B-xyn11A* | Clo1313_0522 | **cgaC**tt**AAA** | 14 (N) | **CGAA**tag**AT** | 169 |
| *Ct* ATCC 27405 | *xyn11A* | Cthe_2972 | **cgaC**tt**AAA** | 14 (N) | **CGAA**tag**AT** | 173 |
| *Ct* DSM 2360 | *xyn11B-xyn11A* | EEU01951.1 | **cgaC**tt**AAA** | 14 (N) | **CGAA**tag**AT** | 170 |
| *Ct* YS | *xyn11B-xyn11A* | YSBL_2860 | **cgaC**tt**AAA** | 14 (N) | **CGAA**tag**AT** | 169 |
| *Ct* AD2 | *xyn11B-xyn11A* | AD2_2992 | **cgaC**tt**AAA** | 14 (N) | **CGAA**tag**AT** | 169 |
| *Ct* JW20 | *xyn11A* | Cther_0345 | **cgaC**tt**AAA** | 14 (N) | **CGAA**tag**AT** | 169 |
| *Ct* BC1 | *xyn11B-xyn11A* | CTHBC1_2886 | **cgaC**tt**AAA** | 14 (N) | **CGAA**tag**AT** | 173 |
| *Cs* JCM 21531 | *xyn11B-xyn11A* | JCM21532_4047_Downstream | **cgaC**tt**AAA** | 14 (N) | **CGAA**tat**AT** | 151 |
| **σ$^{I6}$ promoter consensus** | | | **C--AAA** | **14 (N)** | **CGAA---AT** | |

Bases that match the σ$^{I6}$ promoter consensus are shown in bold capital fonts. *Ct, C. thermocellum*; *Cs, C. straminisolvens*.

[a] Clo1313 and Cthe are the locus tag prefixes of *C. thermocellum* DSM 1313 and ATCC 27405 respectively.
